# Disease-associated missense mutations in the pore loop of polycystin-2 alter its ion channel function in a heterologous expression system

**DOI:** 10.1016/j.jbc.2024.107574

**Published:** 2024-07-14

**Authors:** Tobias Staudner, Linda Geiges, Juthamas Khamseekaew, Florian Sure, Christoph Korbmacher, Alexandr V. Ilyaskin

**Affiliations:** Friedrich-Alexander-Universität Erlangen-Nürnberg, Institute of Cellular and Molecular Physiology, Erlangen, Germany

**Keywords:** autosomal dominant polycystic kidney disease (ADPKD), *renal physiology*, polycystin, *transient receptor potential channels (TRP channels)*, *electrophysiology*, two electrode voltage clamp, *molecular dynamics*, *site-directed mutagenesis*, *Xenopus*, *oocyte*

## Abstract

Polycystin-2 (PC2) is mutated in ∼15% of patients with autosomal dominant polycystic kidney disease (ADPKD). PC2 belongs to the family of transient receptor potential (TRP) channels and can function as a homotetramer. We investigated whether three disease-associated mutations (F629S, C632R, or R638C) localized in the channel’s pore loop alter ion channel properties of human PC2 expressed in *Xenopus laevis* oocytes. Expression of wild-type (WT) PC2 typically resulted in small but measurable Na^+^ inward currents in the absence of extracellular divalent cations. These currents were no longer observed when individual pore mutations were introduced in WT PC2. Similarly, Na^+^ inward currents mediated by the F604P gain-of-function (GOF) PC2 construct (PC2 F604P) were abolished by each of the three pore mutations. In contrast, when the mutations were introduced in another GOF construct, PC2 L677A N681A, only C632R had a complete loss-of-function effect, whereas significant residual Na^+^ inward currents were observed with F629S (∼15%) and R638C (∼30%). Importantly, the R638C mutation also abolished the Ca^2+^ permeability of PC2 L677A N681A and altered its monovalent cation selectivity. To elucidate the molecular mechanisms by which the R638C mutation affects channel function, molecular dynamics (MD) simulations were used in combination with functional experiments and site-directed mutagenesis. Our findings suggest that R638C stabilizes ionic interactions between Na^+^ ions and the selectivity filter residue D643. This probably explains the reduced monovalent cation conductance of the mutant channel. In summary, our data support the concept that altered ion channel properties of PC2 contribute to the pathogenesis of ADPKD.

Autosomal-dominant polycystic kidney disease (ADPKD) is the most frequent hereditary kidney disease and the fourth most prevalent cause of end-stage renal disease (ESRD). It is characterized by the progressive development of fluid-filled renal cysts which disrupt nephron architecture and impair renal function (1, 2, 3). The vast majority of ADPKD cases is caused by mutations in the gene *PKD1* or *PKD2* coding for polycystin-1 (PC1) or polycystin-2 (PC2, also known as TRPP2 or TRPP1), respectively. Germ-line mutations affect PC1 in ∼85% and PC2 in ∼15% of patients with ADPKD. For cyst development, a second somatic mutation is required. Although both proteins were discovered more than 25 years ago (4, 5), their physiological functions as well as pathomechanisms involved in ADPKD remain elusive.

PC2 is a member of the transient receptor potential (TRP) family of non-selective cation channels (6, 7). Initial reports indicated that PC2 requires an association with PC1 to form a fully functional ion channel (8, 9). Moreover, a recently published cryo-electron microscopy (cryo-EM) structure (10) and a subsequent functional study in the oocyte expression system (11) suggest that PC2 and PC1 can form a heteromeric complex with a 3:1 stoichiometry. However, recent studies also support the concept that PC2 can function as a homotetrameric ion channel independently of PC1 (12, 13, 14). Importantly, electrophysiological recordings from excised primary cilia, the dysfunction of which is linked to cystogenesis (15, 16, 17, 18, 19), indicate that PC2 does not require the presence of PC1 to form a ciliary ion channel (13). This is plausible because PC2 is expressed stably during nephrogenesis and in the adult kidney, while PC1 expression is barely detectable in adult kidneys (20). Thus, PC2 malfunction in primary cilia may play a central role in the pathophysiology of ADPKD. In addition, PC2 has also been detected in the plasma membrane and the endoplasmic reticulum (ER) (21, 22, 23, 24, 25, 26). Interestingly, recent findings suggest that PC2 functions as a K^+^ channel mediating potassium-calcium counter ion exchange in the context of agonist-induced ER Ca^2+^ release. Thus, disturbed PC2 function in the ER may contribute to the pathogenesis of ADPKD (27). Taken together, it is currently well established that PC2 has an ion channel function. However, there is still controversy in the literature regarding the role of an altered PC2 ion channel function in the pathogenesis of ADPKD.

Recently published cryo-EM structures demonstrated that PC2 forms homotetrameric complexes (28, 29, 30, 31). Individual subunits are composed of six transmembrane domains (S1-S6), a large extracellular polycystin-specific TOP (tetragonal opening of polycystins) domain localized between S1 and S2, and intracellular N- and C-termini. Importantly, each protomer contributes to the formation of the channel pore by its S5 and S6 domains, which are connected by a pore loop (approx. 34 amino acid residues) from the extracellular side (28, 29, 30, 31). The pore loop includes two pore helices (PH1 and PH2) and the channel’s selectivity filter (_641_LGD_643_). Therefore, the pore loop is likely to play an essential role in ion permeation through the channel. Indeed, in a recent publication (32) we demonstrated that replacing the pore loop of PC2 with that of a related cation channel, polycystin-2-like 1 (PC2L1), changed the ion channel properties of PC2 and initiated a mild form of cystogenesis in a mouse model. Interestingly, a cluster of three ADPKD-associated missense mutations (F629S, C632R, and R638C) located in PH1 of the PC2 pore loop is described in the ADPKD mutation database (http://pkdb.mayo.edu). It has been reported that these mutations have a complete loss-of-function effect when introduced in a constitutively active PC2 construct with an artificial gain-of-function (GOF) mutation, F604P (12, 33). However, how these mutations affect PC2 ion channel function at the molecular level remains incompletely understood.

In this study, we used the *Xenopus laevis* oocyte expression system to investigate the functional effects of these three pore mutations (F629S, C632R, and R638C) on wild-type PC2 (PC2 WT) and on two additional PC2 constructs with previously described GOF mutations, PC2 F604P (12, 33) and PC2 L677A N681A (11). We confirmed a loss-of-function effect of the three pore mutations on PC2 F604P and demonstrated a similar effect on PC2 WT. Importantly, only C632R abolished currents mediated by PC2 L677A N681A, whereas ion channel function was partially preserved with the pore mutations F629S or R638C. Residual channel function was particularly prominent with R638C which also altered ion selectivity of the channel. Molecular mechanisms involved in the inhibitory effect of R638C on ion permeation through the selectivity filter of PC2 were further explored using a combination of electrophysiological measurements, site-directed mutagenesis, and molecular modeling. Taken together, our findings suggest that disease-associated pore mutations of PC2 not simply cause a loss-of-function effect, but may result in residual channel function with altered ion channel properties. This strengthens the concept that, at least in a subset of patients, ADPKD is a channelopathy with impaired PC2 ion channel function.

## Results

### Analysis of polycystin-2 (PC2) structure suggests that the pore mutations F629S, C632R and R638C affect both the channel’s selectivity filter and its gating mechanism

**Figure 1:**
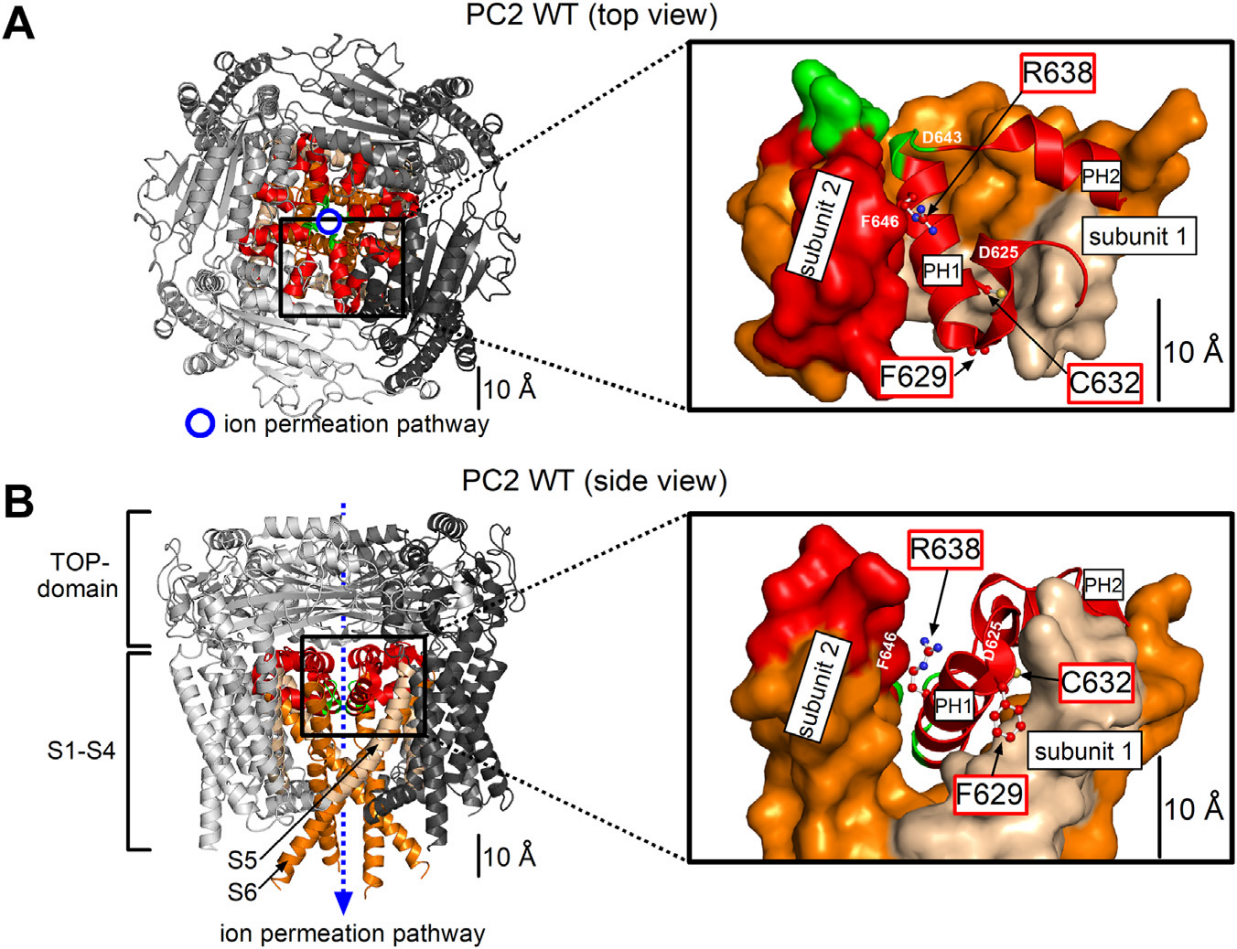
**Localization of three polycystin-2 pore loop residues (F629, C632, R638) mutated in ADPKD.***Top* (*A*) and side (*B*) view of human wildtype polycystin-2 (PC2 WT) homotetramer in ribbon representation generated using atom coordinates from PDB entry 6T9N (35). Individual protomers are colored in different shades of *gray*, and transmembrane domains S5 and S6 of all protomers are shown in *beige* and *orange*, respectively. The pore loop is in *red*, except for three selectivity filter residues (641-LGD-643), which are in *green*. The insets show a portion of PC2 on an expanded scale, where the pore loop of subunit 1 (shown in ribbon representation) forms multiple intra- and intersubunit contacts with several other PC2 domains of subunit 1 and 2 shown in surface representation. Three pore loop residues mutated in ADPKD (F629, C632, and R638) are localized in the pore helix 1 (PH1) and their side chains are shown in ball-and-stick representation with carbon atoms in *red*, nitrogen in *blue*, and sulfur in *yellow*. Hydrogen atoms are omitted for clarity.

The pore loop consists of a pore helix 1 and 2 (PH1 and PH2, respectively). All three ADPKD-associated pore mutations (F629S, C632R, R638C) are localized within the PH1 (Fig. 1, *A* and *B*). Therefore, these mutations may affect ion permeation through the channel’s selectivity filter. Interestingly, PH1 forms tight interactions with S5 and S6 domains of the same PC2 subunit and also intersubunit interactions with PH2 and S6 domains of the neighboring subunit (Fig. 1, *A* and *B*). Thus, mutations in PH1 may also impact channel gating which is associated with conformational changes in S5 and S6 domains.

The aromatic phenylalanine residue F629 is located at the very proximal end of PH1, where it forms, together with several hydrophobic residues of S5, a hydrophobic surface facing membrane lipids (29, 31). Introducing a polar serine residue at this position (F629S) is likely to affect PC2-lipid interactions. This may disturb the pore loop architecture, ion permeation through the selectivity filter, and PC2 gating.

The cysteine residue (C632) is buried within the protein between the proximal end of the pore loop, the distal part of S5, and the proximal part of the S6 domain of the same PC2 subunit. Due to spatial limitations, a large and positively charged arginine side chain would be energetically unfavorable at this position. Thus, the mutation C632R is expected to cause a severe disturbance of the pore loop structure likely to affect both the passage of ions through the selectivity filter and/or PC2 gating.

Finally, the side chain of R638 probably forms interactions with a pore loop residue D625 and a selectivity filter residue D643 in the same PC2 subunit, and also intersubunit interactions with a PH2 residue of the neighboring subunit (F646). The former interactions are likely to be critical for ion conductance of the selectivity filter, whereas the latter may be involved in mediating channel opening (29). Importantly, the R638C substitution probably eliminates these intra- and intersubunit interactions, suggesting that the mutant channel has an altered selectivity filter and disturbed gating.

Taken together, analysis of the PC2 structure indicates that the three pore mutations are likely to have complex and distinct effects on the ion channel properties of PC2, affecting both the selectivity filter and the channel gating.

### Pore mutations F629S, C632R and R638C exhibit a loss-of-function effect on wildtype PC2

**Figure 2:**
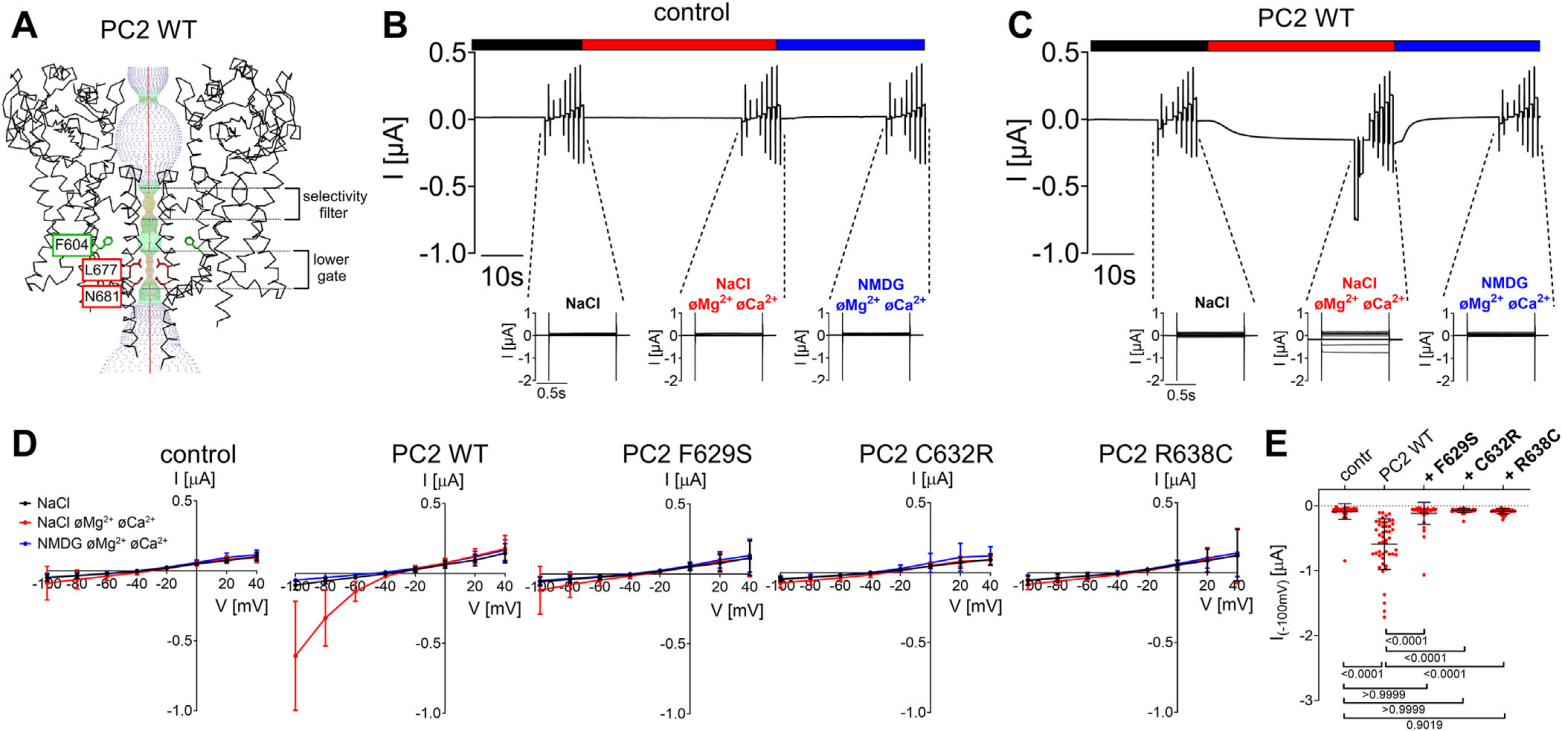
**Pore mutations F629S, C632R and R638C exhibit a loss-of-function effect on PC2 WT.***A*, profile of the ion permeation pathway in PC2 WT generated using the HOLE program (60) is shown along with two diagonally opposed protomers in *black* wire representation. Analysis was made using the PDB entry 6T9N (35). Two apparent pathway constrictions are located at the level of the selectivity filter, and the *lower gate* as indicated. With an intact S5 residue F604 (in sticks representation and in *green*), the narrowest part of the pathway is formed by L677 and N681 side chains shown in sticks representation and in *red* (the lower gate). *B* and *C*, representative whole-cell current traces obtained in a control *Xenopus laevis* oocyte (*B*) or an oocyte injected with 15 ng cRNA encoding human PC2 WT (*C*). Application of different bath solutions (a standard NaCl solution with or without divalent cations (øMg^2+^ øCa^2+^) or an NMDG^+^ solution without divalent cations) is indicated by *black*, *red*, and *blue bars*, respectively. For each condition, voltage step protocols were performed with consecutive 1000 ms voltage steps in 20 mV increments starting with a hyperpolarizing pulse to −100 mV from a holding potential of −60 mV. Overlays of the corresponding whole-cell current traces are shown below the traces. *D*, average I/V-plots (mean ± SD) were constructed from similar recordings as shown in (*B*) and (*C*) and from those obtained in oocytes expressing the PC2 pore mutants (F629S, C632R, R638C) using a similar experimental protocol as in (*B* and *C*). In each recording, the current values measured during the final 300 ms of each pulse were used to construct the corresponding I/V-plot (N = 5, 40 ≤ *n* ≤ 48; N indicates the number of different batches of *Xenopus laevis* oocytes, and *n* indicates the number of individual oocytes analyzed per experimental group). *E*, summary data of the same experiments as shown in (*D*). The maximal inward currents reached during the application of hyperpolarizing pulses of −100 mV in divalent free NaCl (NaCl øMg^2+^ øCa^2+^) bath solution are shown. The *p*-values were calculated by the Kruskal-Wallis test with Dunn’s *post hoc* test.

To investigate the functional effect of the pore mutations on PC2-mediated currents, F629S, C632R, and R638C substitutions were introduced into human PC2 WT. Available structures of PC2 WT show the channel in a closed state (Fig. 2*A*), characterized by a prominent constriction of the ion permeation pathway at the so-called lower gate, which is formed by the side chains of L677 and N681. WT or mutant PC2 channels were heterologously expressed in *X. laevis* oocytes and their functions were assessed by the two-electrode voltage clamp technique. The experimental design was essentially the same as reported previously, *i.e.* divalent cations (Ca^2+^ and Mg^2+^) were removed from the bath solution to elicit PC2-mediated inward Na^+^ currents (11, 12, 14, 32). Subsequently, Na^+^ in the bath was replaced by a large organic cation NMDG^+^ to demonstrate that the inward current component activated by divalent cation removal was carried by Na^+^. In control oocytes without PC2 expression (Fig. 2, *B*, *D* and *E*), simultaneous Ca^2+^ and Mg^2+^ removal as well as subsequent NMDG^+^ application had no detectable effect on the magnitude of the inward currents consistent with our previous observations (32). This indicated that the Na^+^ conductance of control oocytes was negligible. In contrast, in oocytes expressing PC2 WT the simultaneous removal of Ca^2+^ and Mg^2+^ ions elicited a significant inward current component, which was completely abolished by subsequent replacement of bath Na^+^ by NMDG^+^ (Fig. 2, *C*–*E*). However, the magnitude of these currents was very small. This is consistent with previous functional observations and the assumption that baseline activity of PC2 WT at the cell surface of *X. laevis* oocytes is rather low (11, 12, 14, 32). Importantly, currents measured in oocytes expressing PC2 with F629S, C632R or R638C pore mutation were not significantly different from those measured in control oocytes (Fig. 2, *D* and *E*). Analysis of cell surface and intracellular protein expression of PC2 constructs did not reveal a substantial difference between mutant and WT channels (Fig. S1). Therefore, the absence of detectable Na^+^ inward currents in oocytes expressing PC2 with F629S, C632R, or R638C pore mutations could not be attributed to a lack of PC2 expression at the cell surface. Thus, these findings are consistent with a loss-of-function effect of these pore loop mutations on WT PC2. However, we cannot exclude the possibility that the mutant channels have a minor residual function below the detection limit of our current measurements. Therefore, we performed additional experiments to investigate the effects of these pore mutations on PC2 channel constructs with increased constitutive baseline activity due to gain-of-function mutations.

### Pore mutations F629S, C632R and R638C exhibit a loss-of-function effect on PC2 with a known gain-of-function (GOF) mutation (F604P) in the S5 domain

**Figure 3:**
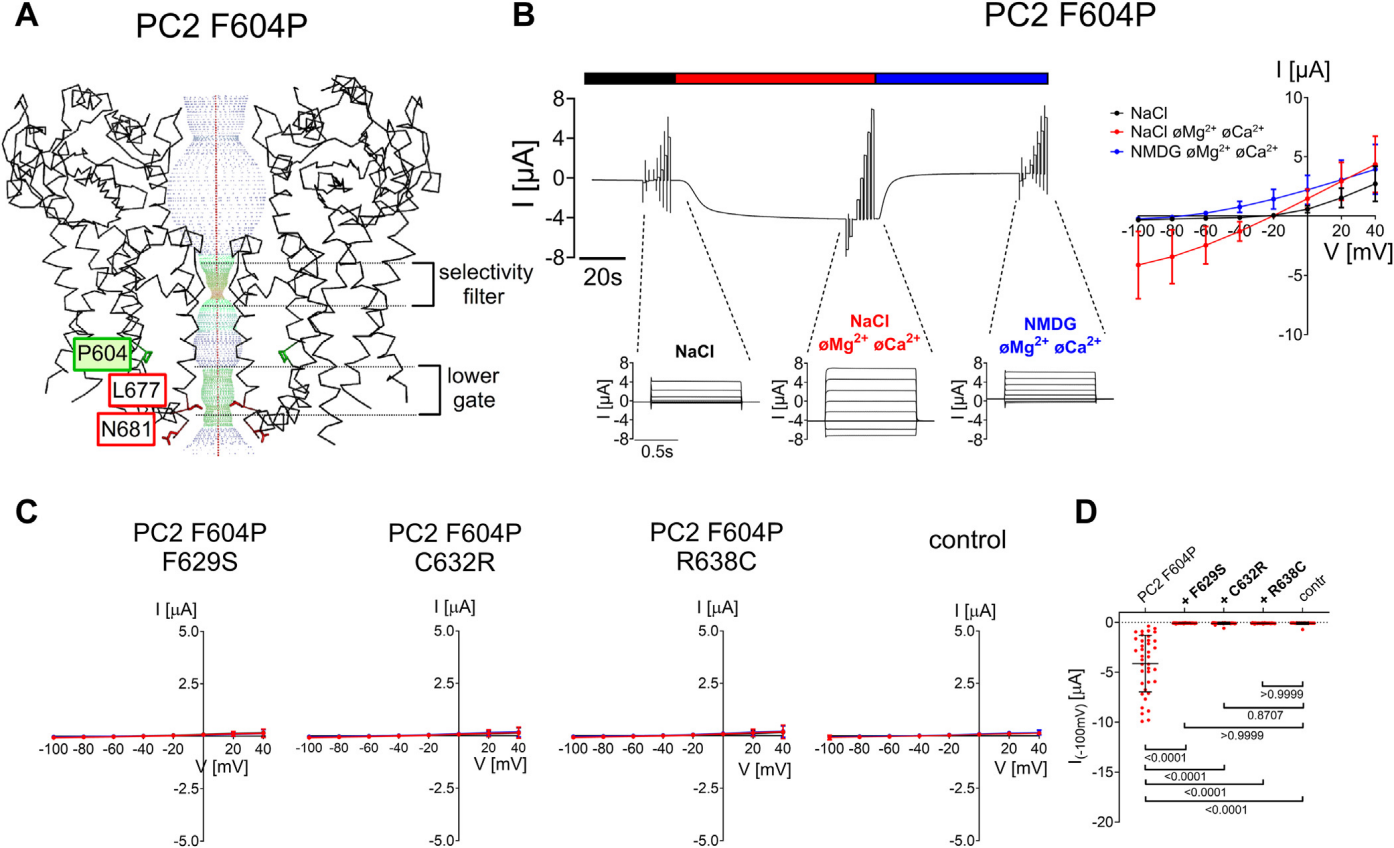
**Pore mutations F629S, C632R and R638C exhibit a loss-of-function effect on PC2 F604P.***A*, profile of the ion permeation pathway in PC2 F604P generated using the HOLE program (60) is shown along with two diagonally opposed protomers in *black* wire representation. Analysis was made using the PDB entry 6D1W (61). It is noteworthy that the F604P mutation (shown in sticks representation and in *green*) removes the pathway constriction at the level of the lower gate by altering the position of L677 and N681 residues. *B*, representative whole-cell current trace obtained in a *Xenopus laevis* oocyte injected with 7.5 ng cRNA encoding human PC2 F604P is shown to the left of the corresponding average I/V-plot (mean ± SD) obtained from recordings using a similar protocol as described in Figure 2. *C*, average I/V-plots (mean ± SD) were obtained from oocytes expressing PC2 with pore mutations (F629S, C632R, R638C) introduced into PC2 F604P and from control oocytes using a similar experimental protocol as in (*B*) (N = 3, 33 ≤ *n* ≤ 39; N indicates the number of different batches of *Xenopus laevis* oocytes, and *n* indicates the number of individual oocytes analyzed per experimental group). *D*, Summary data of the same experiments as shown in (*C*). The maximal inward currents reached during the application of hyperpolarizing pulses of −100 mV in divalent free NaCl (NaCl øMg^2+^ øCa^2+^) bath solution are shown. The *p*-values were calculated by the Kruskal-Wallis test with Dunn’s *post hoc* test.

In the previously identified PC2 F604P GOF construct a phenylalanine residue in the channel’s S5 domain is replaced by a proline residue (12, 14). This F604P substitution is thought to trigger a conformational change of the S6 domain, the so-called π-α-switch. This probably results in the translocation of the lower gate residues L677 and N681 away from the ion permeation pathway and opens the channel’s lower gate (Fig. 3*A*).

In the present study, we could reproduce the reported GOF effect of F604P on PC2 activity. Indeed, in PC2 F604P expressing oocytes, the magnitude of Na^+^ inward currents upon divalent cation removal from the bath solution (Fig. 3, *B* and *D*) was strongly increased in comparison with PC2 WT mediated currents (Fig. 2, *C*–*E*). It is noteworthy that the protein expression of PC2 F604P at the cell surface was similar to that of PC2 WT (Fig. S1 *left upper panel*). This indicates that the significantly larger currents observed with PC2 F604P cannot be attributed to differences in channel cell surface expression. This supports the concept that the baseline activity of PC2 WT is very low compared to that of PC2 F604P which is constitutively high probably due to the opening effect of the mutation on the channel’s lower gate.

Importantly, F629S, C632R, and R638C completely abolished the ion channel function of PC2 F604P (Fig. 3, *C* and *D*), without having a significant effect on its intracellular or cell surface protein expression (Fig. S2). These findings are in good agreement with previous reports (12, 33) and at first sight support the conclusion that the pore mutations have a complete loss-of-function effect on PC2. Alternatively, the pore mutations may disturb the specific conformational change mediating the gain-of-function effect of the F604P substitution. To test this, we used a second gain-of-function PC2 construct which is thought to increase PC2 activity by a different molecular mechanism.

### Ion channel function of PC2 with gain-of-function mutations in the lower gate (L677A N681A) is partially preserved with the pore mutations F629S or R638C but not with C632R

**Figure 4:**
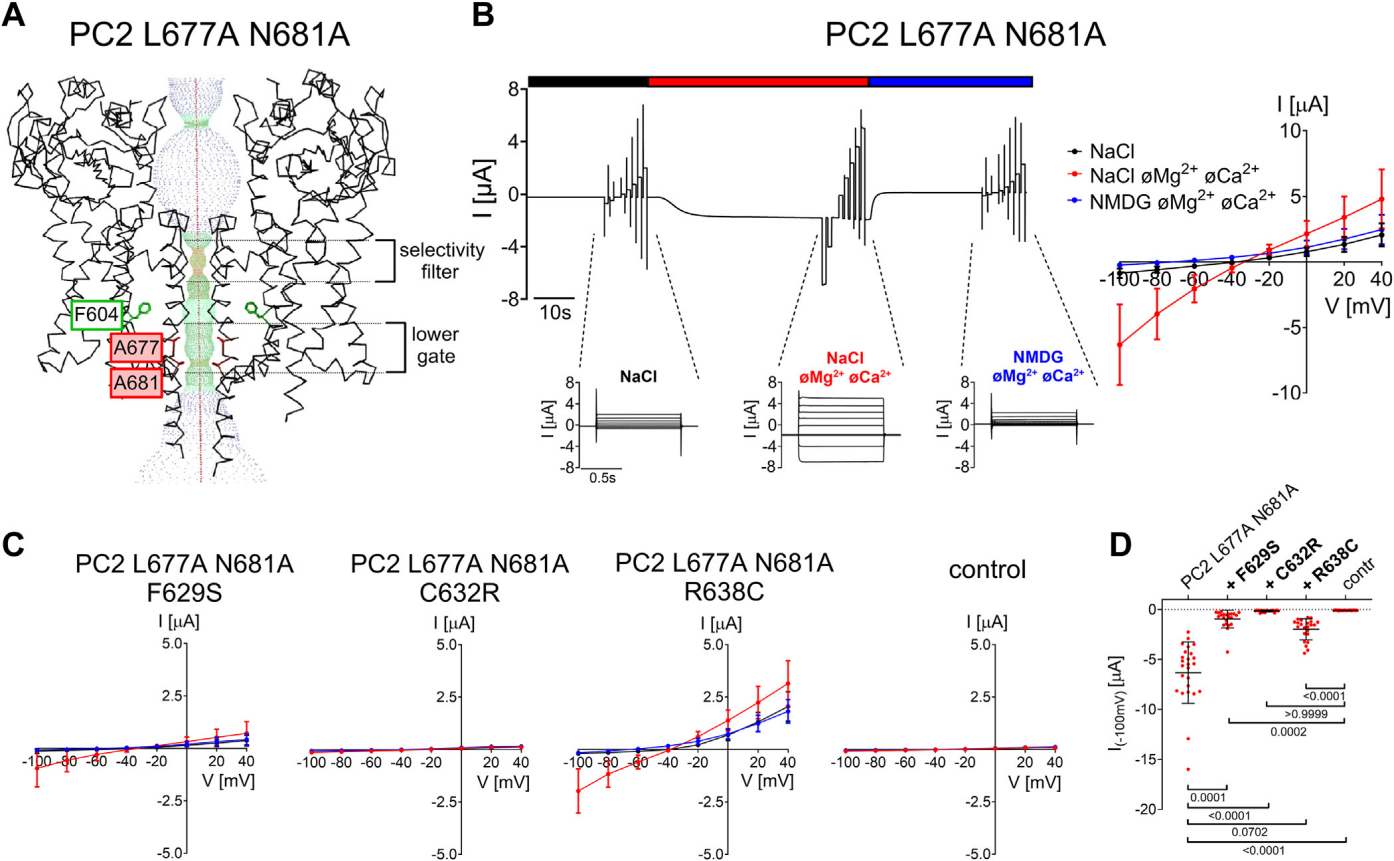
**Na**^**+**^**permeability of PC2 L677A N681A is partially preserved in F629S and R638C mutants, but not in C632R.***A*, profile of the ion permeation pathway in PC2 L677A N681A generated using the HOLE program (60) is shown along with two diagonally opposed protomers in *black wire* representation. Two alanine point mutations (shown in sticks representation and in *red*) were introduced into the PC2 WT [PDB entry 6T9N (35)] *in silico* to remove the pathway constriction at the level of the lower gate. *B*, representative whole-cell current trace obtained in a *Xenopus laevis* oocyte injected with 7.5 ng cRNA encoding human PC2 L677A N681A is shown to the left of the corresponding average I/V-plot (mean ± SD) obtained from recordings using a similar protocol as described in Figure 2. *C*, average I/V-plots (mean ± SD) were obtained from oocytes expressing PC2 with pore mutations (F629S, C632R, R638C) introduced into PC2 L677A N681A and from control oocytes using a similar experimental protocol as in (*B*) (N = 3, 22 ≤ *n* ≤ 25; N indicates the number of different batches of *Xenopus laevis* oocytes, and *n* indicates the number of individual oocytes analyzed per experimental group). *D*, summary data of the same experiments as shown in (*C*). The maximal inward currents reached during the application of hyperpolarizing pulses of −100 mV in divalent free NaCl (NaCl øMg^2+^ øCa^2+^) bath solution are shown. The *p*-values were calculated by the Kruskal-Wallis test with Dunn’s *post hoc* test.

In the established gain-of-function construct PC2 L677A N681A two residues in the lower gate are replaced by alanines (11, 14). These substitutions probably remove the constriction of the ion permeation pathway at the lower gate (Fig. 4*A*; (11)), thereby producing a channel with a constitutively open ion permeation pathway. As expected from previously published findings (11), we observed large inward Na^+^ currents in PC2 L677A N681A expressing oocytes upon removal of divalent cations from the bath solution (Fig. 4, *B* and *D*). These currents were an order of magnitude larger than those observed with PC2 WT (Fig. 2, *C*–*E*), confirming the strong GOF effect of the lower gate mutations on PC2 ion channel function.

Importantly, the individually introduced ADPKD-associated pore mutations had distinct functional effects on PC2 L677A N681A. With C632R Na^+^ inward currents were essentially abolished. In contrast, the ion channel function of PC2 L677A N681A was partially preserved with F629S and even more with R638C (Fig. 4*C*). Indeed, at a holding potential of −100 mV a significant residual Na^+^ inward current of ∼15% or ∼30% was detected with F629S or R638C, respectively (Fig. 4*D*). Western blot analysis revealed no substantial differences in protein expression between the PC2 L677A N681A constructs tested (Fig. S3). In summary, the C632R mutation had a complete loss-of-function effect on PC2 L677A N681A whereas F629S or R638C reduced but did not abolish PC2 L677A N681A ion channel function. These findings probably reflect distinct molecular effects of the three pore mutations on Na^+^ permeation through the channel’s selectivity filter.

### A conservative replacement C632S is compatible with PC2 ion channel function

**Figure 5:**
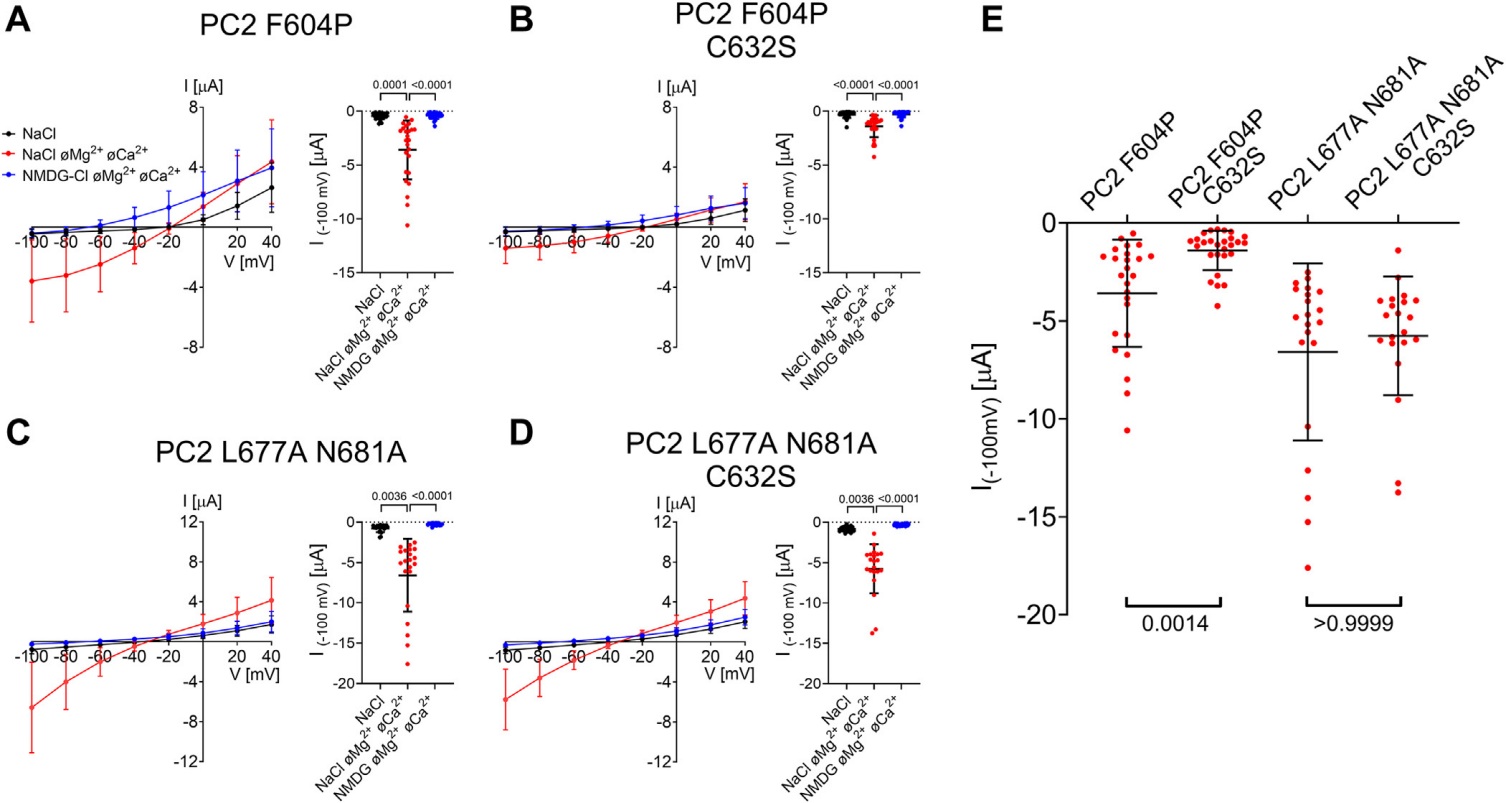
**A cysteine residuein position 632(C632) is not essential for ion channel function of PC2.***A*–*D*, *Left panels*: Average I/V-plots (mean ± SD) were obtained from oocytes expressing PC2 F604P or PC2 L677A N681A without (*A*, *C*) or with cysteine to serine substitution (C632S) (*B*, *D*) using a similar experimental protocol as described is Figure 2. *Right panels*: Summary data show maximal inward currents reached during application of hyperpolarizing pulses of −100 mV in different bath solutions as indicated. Measurements from individual oocytes and mean ± SD are shown (*A*, *B*: N = 2, 25 ≤ *n* ≤ 27; *C*, *D*: N = 2, *n* = 21; N indicates the number of different batches of *Xenopus laevis* oocytes, and *n* indicates the number of individual oocytes analyzed per experimental group; the *p*-values were calculated by Friedman test with Dunn’s *post hoc* test). *E*, summary data of the same experiments as shown in (*A*–*D*). The maximal inward currents reached during the application of hyperpolarizing pulses of −100 mV in divalent free NaCl (NaCl øMg^2+^ øCa^2+^) bath solution are shown. The *p*-values were calculated by the Kruskal-Wallis test with Dunn’s *post hoc* test.

It has been proposed that C632 is essential for PC2 function because it mediates the formation of disulfide bonds between PC2 monomers necessary for PC2 homotetramer assembly (34). Thus, the complete loss-of-function effect of the C632R mutation may be due to an inability of the mutant PC2 monomers to form homotetrameric channels. However, the PC2 structure does not support the concept of direct involvement of C632 in mediating intersubunit interactions (Fig. 1, *A* and *B*). To solve this conundrum by functional studies, we replaced C632 with a serine residue in two PC2 GOF constructs (F604P and L677A N681A) and investigated whether this mutation altered PC2 ion channel function. Cysteine to serine substitution was chosen because it was expected to prevent disulfide bond formation while keeping the size and biophysical properties of the side chain largely unchanged to fit well into the local protein environment (Fig. 1, *A* and *B*). Importantly, and in contrast to the C632R substitution (Figs. 3*C* and 4*C*), the conservative side chain replacement (C632S) did not result in a loss of ion channel function (Fig. 5). Indeed, C632S reduced but did not abolish the currents mediated by PC2 F604P (Fig. 5, *A*, *B* and *E*). Moreover, C632S mutation had no significant effect on PC2 L677A N681A currents (Fig. 5, *C*–*E*). These findings are consistent with the structural data and indicate that C632 is not essential for PC2 homotetramer assembly and function.

### Pore mutation R638C but not F629S alters monovalent cation selectivity of PC2 L677A N681A

**Figure 6:**
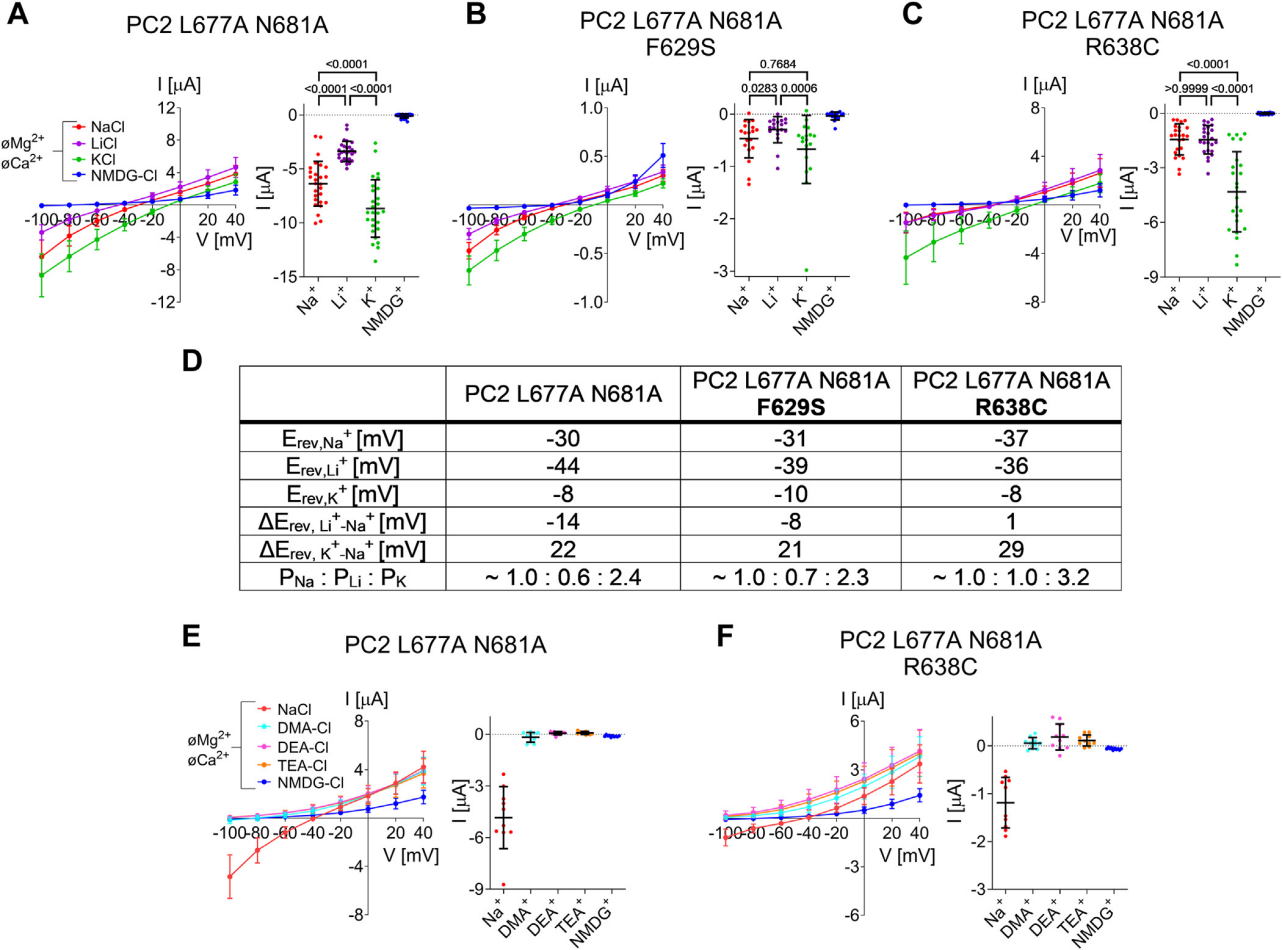
**Pore mutation R638C, but not F629S, alters the selectivity of PC2 L677A N681A for monovalent cations.***A*–*C*, Selectivity for small inorganic monovalent cations was assessed for PC2 L677A N681A (*A*), PC2 L677A N681A F629S (*B*), and PC2 L677A N681A R638C (*C*) by replacing Na^+^ in the bath solution by K^+^, Li^+^ or NMDG^+^ in the absence of divalent cations. *Left panels*: Average I/V-plots (mean ± SD) were obtained using a similar experimental approach as shown in Figure 2. To correct for endogenous oocyte currents (in particular K^+^ currents), the average whole-cell current values measured in control oocytes (Fig. S4*A*) were subtracted from the corresponding individual whole-cell current values measured in oocytes from the same batch expressing PC2 constructs. *Right panels*: Summary data show the maximal inward currents reached during application of hyperpolarizing pulses of −100 mV in the presence of different cations in the bath as indicated. Measurements from individual oocytes and mean ± SD are shown (N = 3, 19 ≤ *n* ≤ 27; N indicates the number of different batches of *Xenopus laevis* oocytes, and *n* indicates the number of individual oocytes analyzed per experimental group; the *p*-values were calculated by repeated measures one-way ANOVA with Bonferroni *post hoc* test (*A*, *C*) or Friedman test with Dunn’s *post hoc* test (*B*)). *D*, reversal potentials (in mV) observed in the presence of Na^+^ (E_rev,Na_^+^), Li^+^ (E_rev,Li_^+^) or K^+^ (E_rev,K_^+^) were estimated from the averaged *I*/*V*-curves shown in (*A*–*C*). Reversal potential shifts were calculated by subtracting E_rev,Na_^+^ from E_rev,Li_^+^ (ΔE_rev, Li_^+^_-Na_^+^) or from E_rev,K_^+^ (ΔE_rev, K_^+^_-Na_^+^) and were used to estimate permeability ratios (P_Na_: P_Li_: P_K_; Equation 1). *E* and *F*, in additional experiments permeability for mid-size organic monovalent cations was assessed for PC2 L677A N681A (*E*) and PC2 L677A N681A R638C (*F*) by replacing Na^+^ in the bath solution by DMA^+^, DEA^+^, or TEA^+^ in the absence of divalent cations. For correction, the values shown in Fig. S4*B* were used. Mean ± SD and individual data points are shown (N = 1, *n* = 10).

In ion substitution experiments we tested whether the pore mutations with preserved ion channel function alter the monovalent cation selectivity of PC2 L677A N681A. Using a similar experimental approach as reported previously (32), we investigated the effect of replacing Na^+^ by Li^+^ or K^+^ on inward currents mediated by PC2 L677A N681A without or with a pore mutation (F629S or R638C) in the absence of extracellular divalent cations (Fig. 6). To correct for endogenous oocyte currents (in particular, for K^+^ currents), the values shown in Figure 6 were corrected by subtracting the average whole-cell current values obtained in the corresponding bath solution in control oocytes from the same batch (Fig. S4*A*). Our findings indicate that without pore mutation PC2 L677A N681A conducted Na^+^ better than Li^+^, and K^+^ better than Na^+^ (K^+^ > Na^+^ > Li^+^; Fig. 6*A*).

Interestingly, the monovalent cation selectivity of PC2 L677A N681A was not qualitatively changed by introducing the F629S mutation (K^+^ > Na^+^ > Li^+^; Fig. 6*B*). In contrast, PC2 L677A N681A with the R638C pore mutation conducted Na^+^ and Li^+^ equally well, whereas its K^+^ conductance was substantially higher than that for Na^+^ or Li^+^ (K^+^ > Na^+^ = Li^+^; Fig. 6*C*). Replacing Na^+^ by K^+^ or Li^+^ caused corresponding reversal potential shifts which were used to estimate cation permeability ratios (Fig. 6*D*). These were in good agreement with the qualitative conclusions reached from the inward current data. The inward currents obtained in K^+^ containing solution were significantly lower in PC2 L677A N681A with F629S or R638C mutation (Fig. 6, *B* and *C*) than in PC2 L677A N681A without pore mutation (Fig. 6*A*). This confirms that F629S and R638C both reduce but do not abolish the function of PC2 L677A N681A as a monovalent cation channel (Fig. 4*C*). In all PC2 constructs, the application of NMDG^+^-containing solution abolished inward currents almost completely, consistent with the absence of a detectable permeability for this large organic cation. In addition, no substantial permeability for mid-sized organic monovalent cations (DMA^+^, DEA^+^, TEA^+^) was observed in oocytes expressing PC2 L677A N681A with or without the R638C mutation (Fig. 6, *E* and *F*). Taken together, our data indicate that the pore mutation R638C, but not F629S, alters the monovalent cation selectivity of PC2 L677A N681A. This result highlights the important role of R638 in the appropriate function of the channel’s selectivity filter.

### No measurable calcium permeability was observed in PC2 L677A N681A with the R638C pore mutation

**Figure 7:**
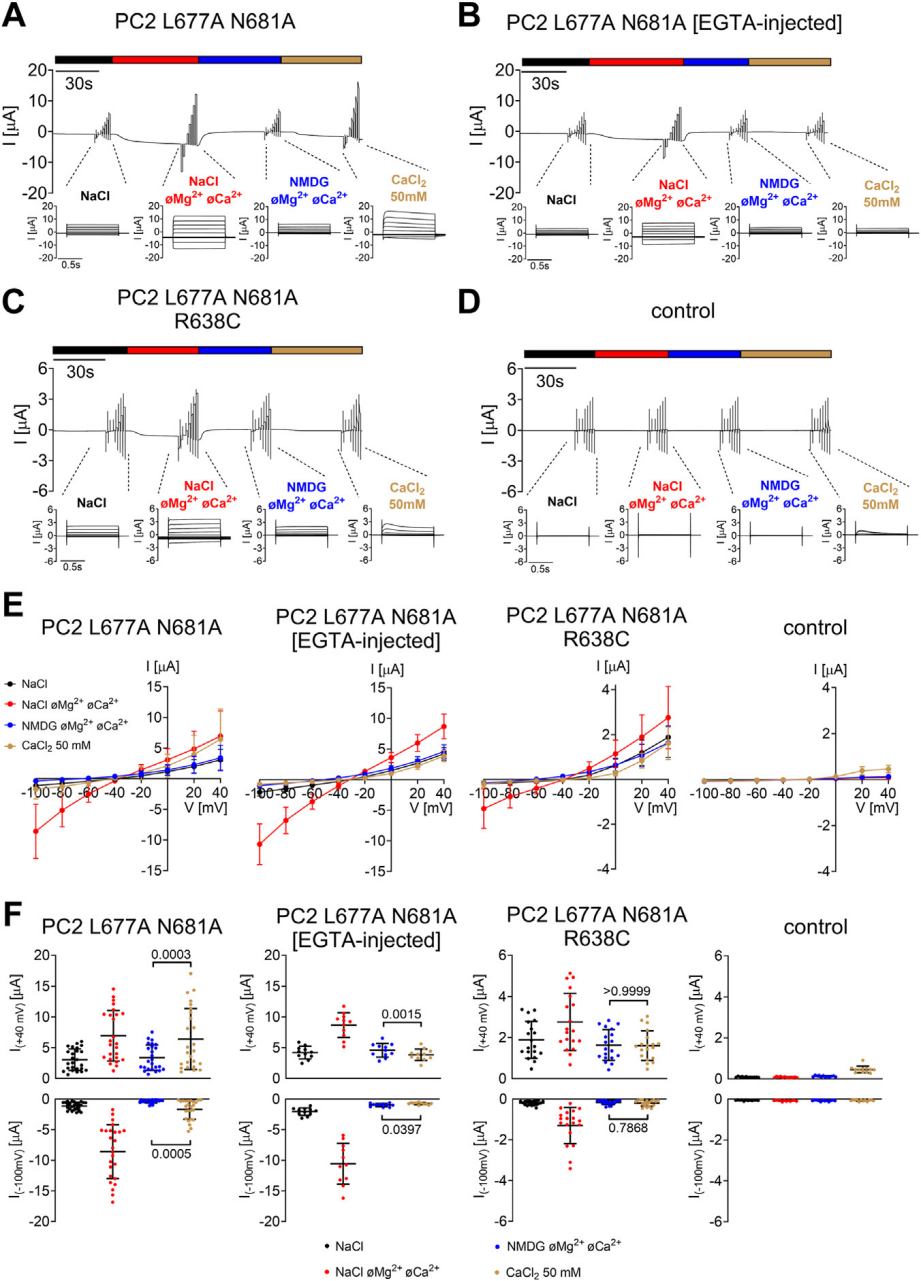
**No measurable calcium permeability was observed in PC2 L677A N681A with R638C pore mutation.***A*–*D*, representative whole-cell current trace obtained in a *Xenopus laevis* oocyte expressing human PC2 L677A N681A without (*A*) or with injection of 50 nl of 50 mM K^+^-EGTA 30 min prior to the measurement (*B*), expressing human PC2 L677A N681A with R638C pore mutation (*C*), or in a control oocyte (*D*). Calcium permeability was assessed by the application of a bath solution containing 50 mM CaCl_2_. *E*, average I/V-plots (mean ± SD) were constructed from similar recordings as shown in (*A*–*D*) (N = 2–3, 12 ≤ *n* ≤ 25; N indicates the number of different batches of *Xenopus laevis* oocytes, and *n* indicates the number of individual oocytes analyzed per experimental group). *F*, summary data of the same experiments as shown in (*A*–*D*). The maximal inward currents reached during the application of depolarizing pulses of +40 mV (*upper panels*) or hyperpolarizing pulses of −100 mV (*lower panels*) in different bath solutions as indicated are shown. The *p*-values were calculated by repeated measures one-way ANOVA with Bonferroni *post hoc* test.

In additional experiments, we investigated whether R638 is essential for Ca^2+^ permeability of PC2. For this purpose, we used PC2 L677A N681A, because for this PC2 construct a substantial Ca^2+^ permeability has been reported (11). Permeability for Ca^2+^ was assessed by applying a 50 mM CaCl_2_ bath solution essentially as described previously (11, 32). In essence, inward currents mediated by Ca^2+^-activated Cl^-^ channels, which are abundantly expressed in the oocyte plasma membrane, were used as a surrogate parameter for PC2-mediated Ca^2+^ influx (11, 32). As expected, PC2 L677A N681A expressing oocytes demonstrated a substantial inward current component in the presence of high extracellular Ca^2+^ concentration (Fig. 7, *A*, *E* and *F*). Moreover, at positive holding potentials large outward currents were observed in 50 mM Ca^2+^ solution, consistent with the stimulation of Ca^2+^-activated Cl^-^ channels. On average, currents measured in high Ca^2+^ solution at −100 mV and at +40 mV were significantly larger than those measured in NMDG^+^-containing solution (Fig. 7*F*). Importantly, prior injection of oocytes with EGTA to chelate Ca^2+^ entering the cell prevented current activation by high extracellular Ca^2+^. This supports the concept that the current activation elicited by high extracellular Ca^2+^ without EGTA injection was due to Ca^2+^-activated Cl^-^ channels (Fig. 7, *B*, *E* and *F*). It is noteworthy that EGTA injection did not disturb PC2 function *per se*, as evidenced by the fully preserved currents measured in Na^+^-containing divalent cation-free extracellular solution (Fig. 7, *B*, *E* and *F*). Our findings confirm the conclusion of a previous study (11) that PC2 L677A N681A is a Ca^2+^ permeable channel, although its Ca^2+^ permeability appears to be rather low compared to its permeability for monovalent cations. Importantly, the R638C mutation completely abolished this Ca^2+^ permeability of PC2 L677A N681A (Fig. 7, *C*, *E*, and *F*). Indeed, PC2 L677A N681A carrying the R638C mutation currents measured in high Ca^2+^ bath solution did not significantly differ from those measured in NMDG^+^-containing bath solution (Fig. 7, *C*, *E* and *F*). In control oocytes application of 50 mM CaCl_2_ did not elicit any substantial inward or outward current response (Fig. 7, *D*–*F*). In summary, these findings confirm that PC2 L677A N681A is a Ca^2+^ permeable channel and reveal that the R638C mutation strongly reduces the Ca^2+^ permeability of the channel. This highlights the functional importance of R638 for normal cation permeation through the channel’s selectivity filter. Moreover, a reduced Ca^2+^ permeability may contribute to the pathophysiological effect of the R638C mutation.

### Computer simulations predict that R638C mutation disturbs a network of side-chain interactions within the pore loop and fosters ionic interactions between Na^+^ and negatively charged pore loop residues

**Figure 8:**
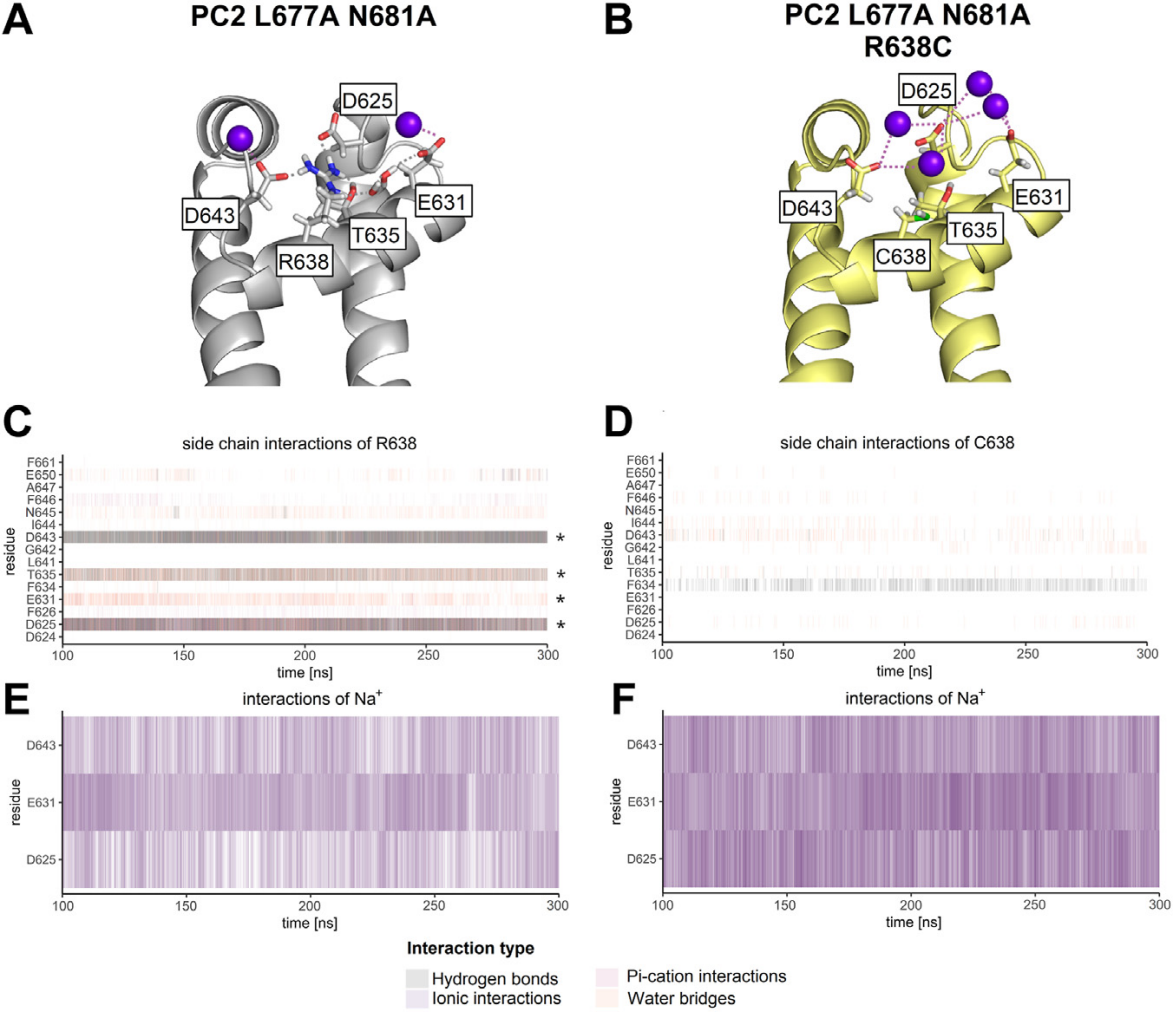
**Atomistic molecular dynamics (MD) simulations suggest that R638C mutation disturbs the network of side-chain interactions within the pore loop and fosters ionic interactions between Na**^**+**^**and negatively charged pore loop residues.***A* and *B*, Representative snapshots taken from the first out of three replica MD simulations of PC2 L677A N681A without (*A*) or with R638C pore mutation (*B*) show key side-chain interactions and ionic interactions with Na^+^ of indicated residues within the pore loop. Ribbon diagrams of the channel’s pore loop are shown with side chains of indicated residues in sticks representation, carbon atoms are in *grey* (*A*) or *yellow* (*B*), hydrogen in *white*, oxygen in *red*, sulfur in *green*, nitrogen in *blue*. Na^+^ are represented as *violet* spheres. *C*–*F*, analysis of interactions was performed over the last 200 ns of the first out of three replica MD simulations of PC2 L677A N681A without (*C*, *E*) or with R638C pore mutation (*D*, *F*). Each vertical line represents the formation of at least one interaction of the specified type per trajectory frame. Interaction types are represented by different colors as indicated. Results from four PC2 subunits are pooled. A darker color intensity corresponds to multiple interactions of the same type observed at the same time point. Key interaction partners of the R638 side chain are highlighted with asterisks in (*C*). Ionic interactions between Na^+^ ions and the negatively charged pore loop residues (D625, E631, D643) are increased due to R638C substitution (*E*, *F*).

To investigate molecular mechanisms involved in the inhibitory effect of the R638C mutation on the cation permeability of PC2, we used atomistic molecular dynamics (MD) simulations. The published PC2 structure [PDB ID: 6T9N (35)] was modified *in silico* by introducing two substitutions at the lower gate (L677A N681A) and one substitution in PH1 (R638C). In control simulations, a corresponding PC2 structure with the lower gate mutations but without the R638C mutation was used. These structures were embedded in a phosphatidylcholine (POPC) bilayer and three replica 300 ns simulations were performed (sim. 1, sim. 2, sim. 3). Importantly, in control simulations we observed that the side chain of R638 formed multiple hydrogen bonds, ionic interactions and water bridges with a cluster of four residues (three negatively charged D625, E631 and D643 and one polar T635) (Fig. 8, *A* and *C*; Fig. S5 and S6). As expected, the cysteine side chain (R638C) failed to form stable interactions with these pore loop residues (Fig. 8, *B* and *D*; Fig. S5 and S6). In contrast, the backbone interactions were largely unaffected by the R638C mutation (Fig. S5 and S7). Three of the four key interacting partners of R638 are negatively charged residues (D625, E631, and D643), which can form ionic interactions with Na^+^ present in the simulation box (Fig. 8*E*). In the absence of positively charged R638, Na^+^ interacted much more frequently with these residues (Fig. 8*F*, Fig. S8 and S9), and the overall interaction time was significantly increased (Fig. S10). Moreover, the R638C mutation resulted in a more stable Na^+^ binding to D625, E631, and D643, as the duration of individual ionic interactions between Na^+^ and these residues appeared to be increased (Fig. S10). In addition, we investigated whether the R638C mutation destabilized the structure of the pore loop. Calculation of RMSF values for side chain atoms did not reveal any differences between the R638C mutant and control, except for the increased flexibility of the D643 side chain (Fig. S11, *A*–*C*). Similarly, RMSF values for backbone atoms were not significantly different in PC2 without or with R638C mutation (Fig. S11, *D*–*F*). Moreover, the dimensions of the selectivity filter, which were defined as the distances between the corresponding backbone atoms L641, G642 and D643 of diagonally opposed PC2 protomers, were similar in PC2 with or without R638C mutation (Fig. S12). These findings argue against the hypothesis, that the arginine to cysteine substitution (R638C) destabilizes the structure of the channel’s pore loop. However, we cannot rule out the possibility, that this mutation may induce a slight but functionally important alteration of the pore loop conformation, which was not revealed by the MD simulations.

To explore the functional role of R638 further, we investigated whether a conservative arginine to lysine substitution (R638K) is compatible with the normal ion channel function of PC2. MD simulations of the R638K mutant revealed that the lysine side chain failed to build the same interaction network with the pore loop residues (Fig. S13, *A* and *B*) as that normally formed by the arginine side chain (Fig. 8, *A* and *C*). In particular, K638 had a reduced ability to form hydrogen bonds with D625, T635, and D643 and water bridges with E631 and T635 (Fig. S13*C*), compared to R638 (Fig. S6). Moreover, we observed more frequent and more stable interactions of Na^+^ with D643 in the R638K mutant, whereas Na^+^ binding to D625 and E631 remained largely unchanged (Fig. S13, *D* and *E*). Importantly, in functional measurements with PC2 L677A N681A carrying the R638K pore mutation, we observed significantly reduced Na^+^ inward currents (Fig. S13*F*) compared to PC2 L677A N681A without this mutation (Figs. 4*B* and 5*C*).

Taken together, these data suggest that even a slightly disturbed network of side-chain interactions within the pore loop in combination with increased binding of Na^+^ to D643 in the selectivity filter results in a significantly decreased Na^+^ permeation through the selectivity filter of PC2.

### Substitution of the negatively charged selectivity filter residue D643 partially rescues the R638C pore mutation and reduces PC2 inhibition by extracellular divalent cations

**Figure 9:**
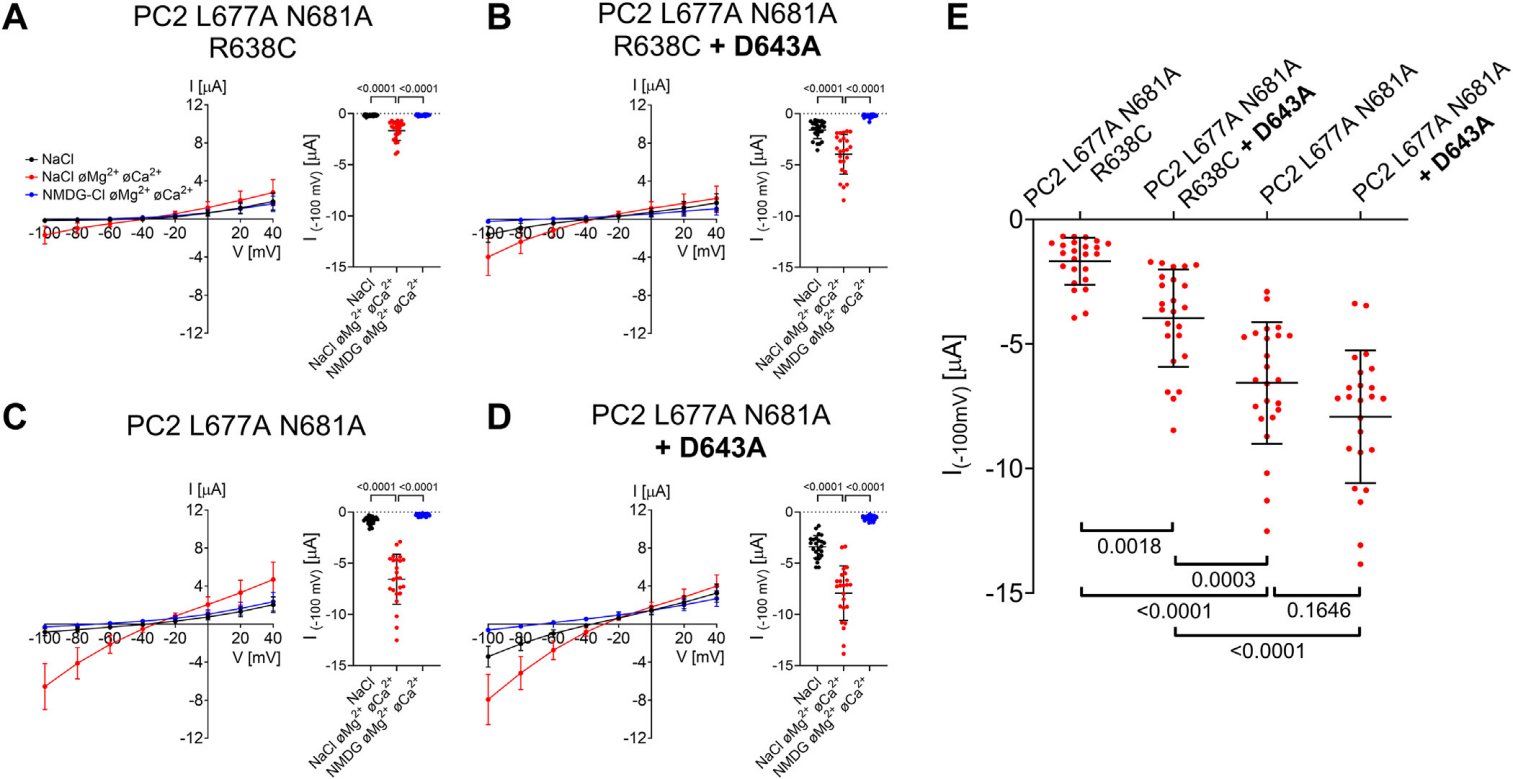
**Mutation of a selectivity filter residue (D643A) partially rescues the Na**^**+**^**conductance of PC2 L677A N681A with R638C pore mutation and reduces channel inhibition by divalent cations (Ca**^**2+**^**, Mg**^**2+**^**).***A*–*D*, *left panels*: Average I/V-plots (mean ± SD) were obtained from oocytes expressing PC2 L677A N681A with R638C pore mutation (*A*), with a combination of R638C and D643A mutations (*B*), without additional mutations (*C*), or with D643A mutation (*D*) using a similar experimental protocol as described in Figure 2. *Right panels*: The summary data show the maximal inward currents reached during the application of hyperpolarizing pulses of −100 mV in different bath solutions as indicated. Measurements from individual oocytes and mean ± SD are shown (N = 2, *n* = 24; N indicates the number of different batches of *Xenopus laevis* oocytes, and *n* indicates the number of individual oocytes analyzed per experimental group; the *p*-values were calculated by repeated measures one-way ANOVA with Bonferroni *post hoc* test). *E*, summary data of the same experiments as shown in *A*–*D*. The maximal inward currents reached during the application of hyperpolarizing pulses of −100 mV in divalent free NaCl (NaCl øMg^2+^ øCa^2+^) bath solution are shown. The *p* values were calculated by one-way ANOVA with the Bonferroni *post hoc* test.

To validate computational predictions further, we tested whether the removal of the negatively charged D643 would produce a rescue effect on PC2 L677A N681A with the R638C pore mutation. Indeed, as shown in Figure 9, *A*, *B* and *E*, introducing the D643A substitution into PC2 L677A N681A with the R638C pore mutation increased Na^+^ inward currents in the absence of divalent cations by more than 2-fold. In contrast, introducing D643A in PC2 L677A N681A without the R638C mutation had only a small stimulatory effect which did not reach statistical significance (Fig. 9, *C*–*E*). It is noteworthy that the D643A substitution also reduced the inhibitory effect of divalent cations (Ca^2+^ and Mg^2+^), as evident from the comparison of the current data obtained in the presence and absence of divalent cations in the bath. In conclusion, these functional data confirm the computational predictions and indicate that the negatively charged D643 has a negative impact on cation permeation through PC2. This effect of D643 is much more prominent in the channel with the R638C pore mutation. In addition, we demonstrated that D643 is partially responsible for PC2 inhibition by extracellular divalent cations.

## Discussion

In this study we investigated the effects of ADPKD-associated PC2 pore loop mutations (F629S, C632R, and R638C) on the function of human PC2 constructs heterologously expressed in *X. laevis* oocytes. We confirmed the previously described loss-of-function effect of these mutations on PC2 with the F604F GOF mutation and demonstrated a similar inhibitory effect on PC2 WT currents. Importantly, currents mediated by another PC2 GOF mutant (L677A N681A) were largely abolished by C632R but merely reduced by F629S and R638C. We provide evidence that the loss-of-function effect of the C632R mutation cannot be attributed to a loss of disulfide bonds between PC2 monomers previously thought to be required for channel assembly. Instead, the introduced arginine residue probably impairs ion permeation through the channel’s selectivity filter. Moreover, our data indicate that R638 critically determines the monovalent cation selectivity and Ca^2+^ permeability of PC2. Finally, using a combination of structure-based simulations with molecular biological methods and electrophysiological measurements, we elucidated molecular mechanisms contributing to the functional roles of R638 and the selectivity filter residue D643 in determining PC2 ion permeation.

In the absence of known specific inhibitors and activators, the characterization of PC2 ion channel function is challenging. This probably explains conflicting findings regarding the electrophysiological properties of PC2 reported in the literature (36, 37). In the current work, we used divalent cation (Ca^2+^, Mg^2+^) removal from the extracellular solution as an established maneuver to activate PC2-mediated currents (12, 32). To minimize the non-specific effects of divalent cation removal, we expressed PC2 in *X. laevis* oocytes in which expression of endogenous connexin 38 (Cx 38) was effectively suppressed using a previously described approach (32, 38, 39, 40). Using this method, we were able to detect small inward currents in PC2 WT expressing oocytes consistent with our previous observations (32). Importantly, we confirmed the previously reported finding (12) that at the cell surface protein expression of PC2 WT was similar to that of the F604P GOF construct. Thus, the small inward currents detected in PC2 WT expressing oocytes suggest that baseline activity of PC2 WT is very low under our experimental conditions, probably because the channel predominantly remains in a closed state. Interestingly, these putative PC2-mediated currents demonstrated pronounced inward rectification, which was not observed in electrophysiological recordings of PC2 in primary cilia (13) and was absent in GOF PC2 constructs as shown in the present study in agreement with previous reports (11, 12). However, a strong inward rectification has been described for mucolipins (TRPMLs) which are non-selective cation channels closely related to PC2 (7). The reason for these discrepant findings is currently unknown, but it is not inconceivable that WT PC2 displays inward rectification under certain experimental conditions. Importantly, each of the three pore mutations (F629S, C632R, and R638C) associated with ADPKD produced a loss-of-function effect on PC2 WT. These findings support our interpretation that the observed inward currents in PC2 WT-expressing oocytes are probably mediated by functional PC2 channels with a low open probability.

As mentioned above, a natural tool to activate WT PC2 in the oocyte expression system is lacking. Therefore, to characterize the functional effects of the pore mutations on PC2 in an open (active) state of the channel, we used two established GOF PC2 constructs (F604P and L677A N681A) (11, 12) despite the obvious limitations of this approach. First, we confirmed a previously published observation that the pore loop mutations produce a loss-of-function effect on PC2 with the F604P GOF mutation (12, 33). A previous structural analysis of this GOF mutant revealed that F604P substitution triggers a switch from π-helix to α-helix in the channel’s sixth transmembrane domain (S6), thereby stabilizing its lower gate in an open conformation (14). All three pore mutations may hinder this stimulatory effect of the F604P mutation on channel gating, thereby causing a loss-of-function effect.

Interestingly, π-helices at similar positions were structurally resolved in several TRP channels and are implicated in their gating (41, 42, 43). Thus, it has been suggested that the conformational change produced by the F604P mutation recapitulates the natural gating mechanism of PC2 (11, 36). However, in TRPML1 and TRPML3, two ion channels closely related to PC2, the π-helix in S6 does not seem to be involved in ligand-mediated channel activation (44, 45, 46). Moreover, the π-helix was not found in structures of PC2L1 channels (47, 48). Thus, we cannot exclude that the gating mechanism of PC2 triggered by the F604P substitution is artificial and differs from that occurring under physiological conditions. Presently, it remains unclear whether the three pore loop mutations affect the natural gating mechanism of PC2.

Due to the complete loss-of-function effect of the three pore mutations on PC2 F604P, the results obtained with this GOF construct provided no information regarding the possible effects of these mutations on ion permeation through the channel’s selectivity filter. Therefore, we went on to characterize the functional effects of these pore mutations on a second PC2 GOF construct (L677A N681A). Structural information on PC2 L677A N681A is lacking, but analysis of PC2 WT structures suggests that this double mutation removes the hydrophobic constriction at the level of the lower gate (14). Thus, the mutated channel probably has a constitutively open ion permeation pathway and does not require active gating to reach an open state. Furthermore, its conductive properties are expected to rely solely on a single constriction produced by the selectivity filter residues (_641_LGD_643_). Therefore, the PC2 L677A N681A construct is probably not suitable to investigate possible effects of ADPKD-associated mutations on the gating mechanism of PC2. However, this construct can be used as a tool to study the impact of ADPKD-associated pore mutations on ion permeation through the channel’s selectivity filter.

We demonstrated that C632R completely abolished PC2 L677A N681A mediated currents, which suggests that this mutation leads to a collapse of the selectivity filter. This finding is consistent with the hypothesis, that an arginine residue, due to its size and positive charge, cannot be tolerated at the 632 position and most likely produces large conformational changes of the pore loop. In contrast, the conservative substitution C632S did not change ion channel properties of PC2 L677A N681A. This latter finding argues against the previously proposed essential role of this cysteine residue in PC2 subunit assembly (34).

Importantly, two other pore mutations (F629S and R638C) did not abolish but merely reduced PC2 L677A N681A currents, which indicates a reduced cation permeability of the channel’s selectivity filter due to these mutations. In addition, we demonstrated that the R638C mutation altered cation selectivity and abolished Ca^2+^ permeability of PC2 L677A N681A. The reduced Ca^2+^ permeability of this mutant channel may be of particular pathophysiological importance because a disturbed intracellular Ca^2+^ balance is generally thought to contribute to the pathogenesis of ADPKD (49). In conclusion, our data obtained with the second GOF PC2 construct (L677A N681A) provided valuable new insights into possible functional effects of the pore mutations on PC2 in its open state. From our experiments it is difficult to predict how the pore mutations may affect naturally gated PC2 channels *in vivo*. However, collectively the results from our functional analysis of three different PC2 constructs (WT; F604P; L677A N681A) support the concept that ADPKD-associated pore mutations impair normal ion channel function of PC2 by affecting its gating and disturbing cation permeation through the channel’s selectivity filter.

The selectivity filter residue D643 is believed to be essential for cation selectivity (12) and conductance of PC2 (29, 36). A circle of four D643 residues (one from each subunit) frames the entrance of the pore and forms the bottom of the channel’s upper vestibule (28, 29). Negatively charged aspartate or glutamate selectivity filter residues are a common feature of the majority of TRP channels (42, 43, 50, 51). The negative charge expels anions and promotes electrostatic accumulation and coordination of permeating cations in the vicinity of the selectivity filter. This concept was supported by the finding, that replacing an aspartate residue at the equivalent position with an alanine (D523A) abolished the ion channel function of PC2L1 (52).

**Figure 10:**
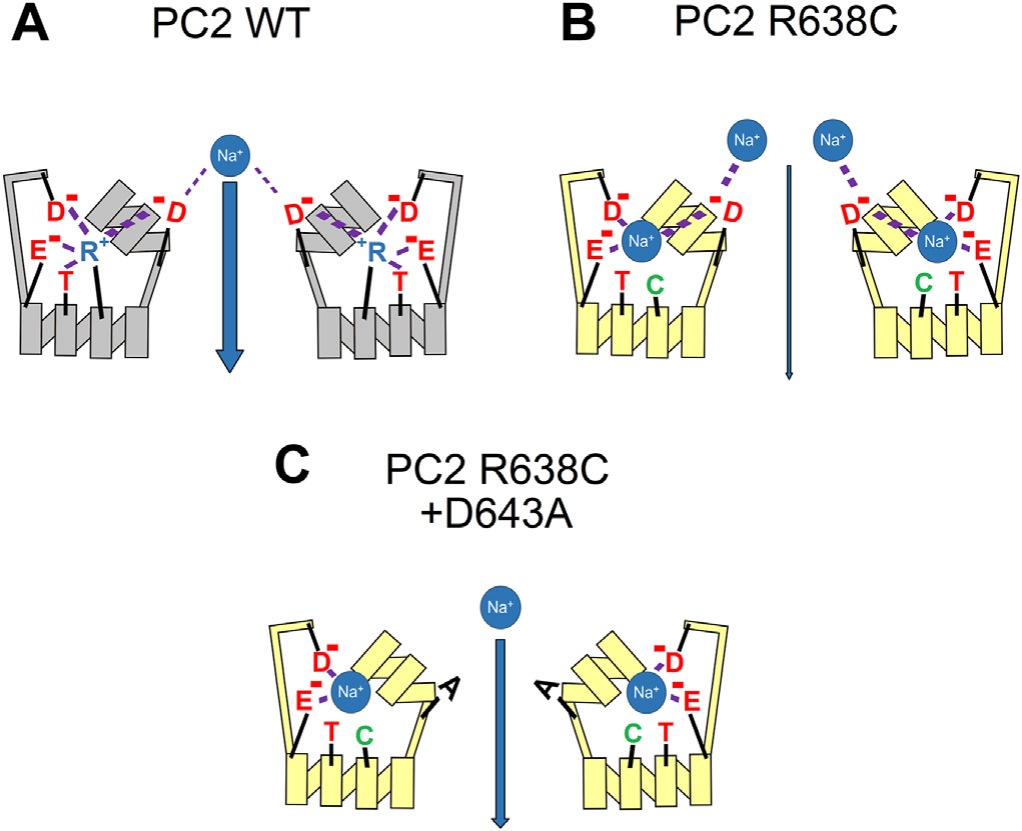
**The putative molecular mechanism underlying the functional effects of the pore loop mutations R638C and D643A on Na**^**+**^**passage through the selectivity filter of PC2.***A*, in WT channel, R638 forms hydrogen bonds, ionic interactions, and water bridges with several pore loop residues (D625, E631, T635, and D643). This probably is essential to support the native conformation of the channel’s pore loop (*gray*). Moreover, spatial limitations and electrostatic repulsion produced by R638 prevent the formation of strong ionic interactions between Na^+^ and a selectivity residue D643, thereby allowing efficient Na^+^ passage through the selectivity filter. *B*, substitution of R638 by a cysteine (R638C) disturbs the delicate network of side-chain interactions within the pore loop, which probably slightly alters its conformation (*yellow*). Moreover, in the absence of the positively charged R638, the negatively charged D625, E631 and D643 increase their capacity to bind Na^+^ and form more stable ionic interactions with Na^+^. Increased electrostatic attraction of Na^+^ to D643 likely impedes dissociation of Na^+^ from D643. Together with the disturbed pore loop conformation, this reduces the Na^+^ conductance of the channel’s selectivity filter. *C*, in contrast, removing the negative charge from the selectivity filter by the D643A substitution increases its Na^+^ conductance. However, this probably cannot restore the native conformation of the pore, which may explain why the D643A substitution only partially rescues Na^+^ permeation through the selectivity filter.

Surprisingly, in our experiments D643A substitution had no negative impact on PC2-mediated currents, indicating that D643 is not essential for cation conductance through the PC2 pore. Importantly, the results of our structure-based MD simulations combined with the functional experiments suggest a new concept of ion permeation through the selectivity filter of PC2, which critically involves a functional interplay between R638, D643 and Na^+^ ions (Fig. 10). Our simulations predicted, that in WT channel R638 forms particularly stable interactions with several pore loop residues (D625, E631, T635, and D643). This interaction network is probably essential for the appropriate conformation of the channel’s pore loop and the selectivity filter. Indeed, previously published structural analysis of PC2 in two different states highlighted an important role of R638 interaction with D643 in determining the pore radius of the channel (30). The arginine-to-cysteine substitution (R638C) or even a more conservative arginine-to-lysine substitution (R638K) disturbs the network of side-chain interactions within the pore loop. This is likely to cause subtle conformational changes of the pore loop and the selectivity filter. These changes could not be resolved by our MD simulations probably due to technical limitations of the method. However, this conclusion is supported by our experimental findings demonstrating an altered monovalent cation selectivity and abolished Ca^2+^ permeability of PC2 L677A N681A with the R638C mutation. Furthermore, our MD simulations predicted that the R638C and R638K substitutions increased the binding capacity of D643 for Na^+^ and the stability of these ionic interactions. We hypothesize that a strong interaction between D643 and Na^+^ impedes dissociation of Na^+^ from D643, thereby reducing Na^+^ permeation through the channel’s selectivity filter. Conversely, in the wildtype channel, the presence of R638 prevents the formation of stable interactions between Na^+^ and D643, thereby facilitating Na^+^ permeation through the selectivity filter. Consistent with this hypothesis, removing the negative charge from the selectivity filter by the D643A substitution partially rescued the Na^+^ conductance of PC2 with the R638C mutation. The finding that the D643A mutation produced only a partial rescue indicates that additional factors like altered conformation of the pore loop may contribute to the inhibitory effect of the R638C mutation. In conclusion, an altered pore loop conformation and increased binding of Na^+^ to D643 are likely molecular mechanisms contributing to the reduced Na^+^ permeability of the R638C mutation observed experimentally. In addition, we demonstrated that D643 is involved in Ca^2+^-mediated inhibition of PC2 as previously postulated from structural analysis (30).

Disturbed ion channel function of several TRP family members results in systemic diseases classified as channelopathies with various cardiac, renal, skeletal, and neurological manifestations (53, 54). The results presented in the current study suggest that impaired ion channel function of PC2 contributes to the pathogenesis of ADPKD at least in a subset of patients with disease-associated PC2 mutations in the channel‘s pore. Moreover, our data indicate that at least one ADPKD-associated pore loop mutation (R638C) may not simply cause a loss-of-function effect, but may result in functional channels with altered ion channel properties. Interestingly, the majority of identified ADPKD-associated missense mutations (http://pkdb.mayo.edu) are localized within the TOP domain of PC2 (28, 36). The TOP domain directly interacts with the channel’s pore region (28, 29, 30, 31, 55) and therefore may play a crucial role in regulating PC2 function as an ion channel. Indeed, Vien *et al.* have recently demonstrated that several ADPKD-associated mutations in the TOP domain inhibit the ion channel function of PC2 in primary cilia (55). Future studies determining the ion channel function of PC2 with disease-causing mutations in the TOP domain may strengthen the concept of ADPKD as a channelopathy. In addition, it will be of interest to assess the functional effects of ADPKD-associated PC2 mutations on the heteromeric PC2/PC1 ion channel. A better understanding of molecular mechanisms by which various ADPKD-associated PC2 mutations affect PC2 or PC2/PC1 ion channel function may promote the development of novel personalized ADPKD treatment strategies.

## Experimental procedures

### Expression plasmids

Full-length complementary DNA (cDNA) encoding human polycystin-2 (PC2) was kindly provided by R. Witzgall (Regensburg, Germany). For heterologous expression in *X. laevis* oocytes, PC2 was subcloned into the pTLN vector (56). N-terminal HA-epitope tag (YPYDVPDYA) and all point mutations were introduced to PC2 using the QuikChange Lightning site-directed mutagenesis kit (Agilent). Sequences of all constructs were routinely verified by a commercially available sequence analysis service (LGC Genomics GmbH, Germany). Plasmids were linearized and used as templates for cRNA synthesis using SP6 RNA polymerase (mMessage mMachine SP6, Ambion).

### Isolation of *X. laevis* oocytes and two-electrode voltage-clamp (TEVC) experiments

Isolation of *X. laevis* oocytes and two-electrode voltage-clamp (TEVC) experiments were essentially performed as described previously (32, 40, 57). Ovarian lobes were excised by partial ovariectomy under anesthesia with Tricain 0.2%, in accordance with the principles of German legislation, with approval by the animal welfare officer for the University of Erlangen-Nürnberg (FAU), and under the governance of the state veterinary health inspectorate. Isolated ovarian lobes were treated with 600 to 700 U/ml collagenase type-2 from *Clostridium hystolyticum* (Sigma-Aldrich) for 3 to 4 h at 19 °C. Defolliculated stage V-VI oocytes were injected with the same amount of cRNA (7.5 ng or 15 ng as indicated in figure legends) encoding a corresponding PC2 construct. To suppress the expression and possible interference of endogenous connexin 38 hemichannels, an established approach was used (32, 38, 39, 40): 3 ng of an antisense phosphothioate oligomer corresponding to nucleotides −5 to +25 relative to the coding region of connexin 38 (5′-GCTTTAGTAATTCCCATCCTGCCATGTTTC-3′) (biomers.net) were co-injected together with PC2 cRNA. Oocytes injected only with the antisense DNA against connexin 38 were used as controls. After cRNA injection, oocytes were incubated in ND9 solution (composition in millimolar: 9 NaCl, 2 KCl, 87 N-methyl-D-glutamine-Cl (NMDG-Cl), 1.8 CaCl_2_, 1 MgCl_2_, 5 HEPES, and pH 7.4 adjusted with Tris) supplemented with 100 units/ml of sodium penicillin and 100 μg/ml of streptomycin sulfate at 19 °C for 72 h (in experiments shown in Fig. 2) or 48 h (in all other experiments). In TEVC experiments, oocytes were continuously superfused with a bath solution as indicated in the figures at an approximate flow rate of 10 ml/min. A modified ND96 solution was used as a standard NaCl bath solution (composition in millimolar: 96 NaCl, 4 KCl, 1 CaCl_2_, 1 MgCl_2_, 10 HEPES, and pH 7.4 adjusted with Tris). To obtain a NaCl bath solution nominally free of divalent cations, CaCl_2_ and MgCl_2_ were excluded from the ND96 solution. Subsequent replacement of 95 mM NaCl with the same concentration of NMDG-Cl resulted in an NMDG bath solution without Na^+^ and divalent cations. To assess the selectivity of PC2 for monovalent cations, bath solutions had the following composition (in millimolar): 100 NaCl, 100 LiCl, 100 KCl, 100 DMA-Cl, 100 DEA-Cl, 100 TEA-Cl, or 100 NMDG-Cl, 10 HEPES, pH 7.4 adjusted with Tris. To estimate the Ca^2+^ permeability of PC2, a bath solution with a high Ca^2+^ concentration was used (composition in millimolar: 50 CaCl_2_, 10 HEPES, and pH 7.4 adjusted with Tris). Bath solution exchanges with a gravity-fed system were controlled by a magnetic valve system (ALA VM8; ALA Scientific Instruments). Measurements were conducted at room temperature using a continuous holding potential of −60 mV. At the end of each solution application, a voltage step protocol was performed as described in the legend in Figure 2. The permeability ratios for monovalent cations (P_Na_: P_K_: P_Li_; Fig. 6*D*) were calculated from the corresponding reversal potential shifts using the modified GHK equation (58):

(1) $${P_{X}}^{+}\;/\;{P_{\text{Na}}}^{+}=e{\;}^{\Delta \text{ErevF}/\text{RT}}$$

where ΔE_rev_ represents the shift of the reversal potential (E_rev_) caused by replacing Na^+^ in the bath solution with cation X^+^ [ΔE_rev_ = E_rev,X_^+^- E_rev,Na_^+^], *F* is the Faraday’s constant, *R* is the universal gas constant, and *T* is the absolute temperature in Kelvin. Data analysis was performed using the programs Microsoft Excel and ‘Nest-o-Patch’ (http://sourceforge.net/projects/nestopatch) written by Dr V. Nesterov (Friedrich-Alexander-Universität Erlangen-Nürnberg, Institute of Cellular and Molecular Physiology, Erlangen, Germany).

### Detection of Polycystin-2 at the cell surface

In parallel to TEVC experiments, cell surface expression of PC2 was assessed using a biotinylation approach essentially as described previously (40, 57, 59). Briefly, oocytes were treated with EZ-Link Sulfo-NHS-SS-Biotin (#21331, Thermo Fisher Scientific) and lysed by mechanical shearing through a needle before biotinylated proteins were precipitated with Pierce NeutrAvidin agarose beads (#29201, Thermo Fisher Scientific). After separation by SDS-PAGE, HA-tagged PC2 was detected on a PVDF membrane using a monoclonal rat anti-HA antibody (clone 3F10; #12158167001, Roche Diagnostics) at a dilution of 1:1000 and a secondary horseradish peroxidase-conjugated goat-anti-rat antibody (#12-035-006, Jackson Immunoresearch) at a dilution of 1:10,000. To confirm the separation of biotin-labeled cell surface proteins from intracellular proteins, western blots were subsequently stripped and reanalyzed using a polyclonal rabbit anti-β-actin antibody (A2066, Sigma-Aldrich) at a dilution of 1:5000 or 1:10,000 and a secondary horseradish peroxidase-conjugated goat-anti-rabbit antibody at a dilution of 1:50,000 (ab6721, Abcam).

### Molecular dynamics (MD) simulations

Point mutations L677A, N681A, R638C, and R638K were introduced *in silico* into a published cryo-EM structure of PC2 [PDB-ID: 6T9N (35)] using Schrödinger Maestro suite version 2021-2. PC2 structure was prepared for MD simulations by adding missing hydrogen atoms and residues and by capping open protein N- and C-termini with N-methyl amide (NME) and N-acetyl groups (ACE), respectively, using the Protein Preparation Wizard. Model systems with simulation box dimensions of approx. 110 × 110 × 120 Å were prepared using 3D-periodic boundaries with the Schrödinger Desmond module. Model systems contained approx. 140,000 atoms including atoms of PC2 embedded into a POPC (phosphatidylcholine) bilayer placed at the level of PC2 transmembrane domains (S1-S6), water molecules (SPC, simple point charge model), 145 mM sodium chloride, and additional sodium counterions to achieve electroneutrality. MD simulations were conducted in an isothermal isobaric (*NPγT*) ensemble with a constant surface tension (γ) of 0 bar × Å imposing 1 atm and 310 K, respectively, using the OPLS4 force field. Three replica 300 ns simulations (sim.1, sim.2, sim. 3) for PC2 L677A N681A and PC2 L677A N681A R638C were performed. Replica simulations differed in the assigned initial velocities of the atoms, which were set randomly. MD trajectory sampling intervals were 100 ps (for sim.1) and 300 ps (for sim.2, sim. 3). In addition, one 300 ns simulation of PC2 L677A N681A R638K was performed with a sampling interval of 100 ps. All molecular dynamics simulations (7 simulations in total) started with the default step-wise relaxation protocol. Subsequently, the temperature was kept constant at 310 K using the Nosé-Hoover thermostat with a relaxation time of 1 ps. The isotropic pressure of 1.013 bar was maintained in the MD system with the Martyna-Tobias-Klein barostat with a relaxation time of 2 ps. The cutoff radius for the Coulomb interactions was set to 9 Å. The integration timestep of a reversible reference system propagator algorithm integrator was set to 2, 2, and 6 fs for bonded, near, and far interactions, respectively. The conformational stability of PC2 during each simulation was analyzed by calculating the root mean square deviations (RMSD) from the starting structure. The first 100 ns of each simulation, during which relaxation of the model system typically occurred, were excluded from further analysis (*i.e.* calculation of root mean square fluctuation (RMSF), analysis of interactions, and measurement of distances). Thus, PC2 parameters were determined for a relatively stable protein conformation. RMSF calculation for backbone and sidechain atoms of each amino acid and interaction analysis was performed using the Schrödinger Simulation Interactions Diagram tool. Measurement of distances was made using the Schrödinger tool for MD trajectory analysis. The duration of interactions between sodium ions and PC2 residues was calculated by a custom-made Python script. The script is available at https://github.com/f-sure/interaction-duration-MD.

### Statistical analysis

N indicates the number of different batches of *X. laevis* oocytes, and *n* indicates the number of individual oocytes analyzed per experimental group. For MD results, n indicates the number of individual data points per group. If not stated otherwise, data are presented as mean ± SD. Statistical evaluation was done using an appropriate statistical test as indicated in figure legends using GraphPad Prism version 10 (GraphPad Software Inc). The normal distribution of data was assessed using the D'Agostino-Pearson omnibus test. Graphical representations were created using the GraphPad Prism version 10, and R environment for statistical computing version 4.0.3 (R Foundation for Statistical Computing; www.R-project.org).

## Data availability

All data are contained within the manuscript and the supporting information.

## Supporting information

This article contains supporting information.

## Conflict of interest

The authors declare that they have no conflicts of interest with the contents of this article.
