# Supplementary material for: Disease-associated missense mutations in the pore loop of polycystin-2 alter its ion channel function in a heterologous expression system
Source: J Biol Chem. 2024 Jul 14;300(8):107574. doi: 10.1016/j.jbc.2024.107574 (PMC11630642; doi:10.1016/j.jbc.2024.107574)
Supplement: Supporting Information [file mmc1.pdf]

Disease-associated missense mutations in the pore loop of polycystin-2 alter its ion channel function in a heterologous expression system

Tobias Staudner, Linda Geiges, Juthamas Khamseekaew, Florian Sure, Christoph Korbmacher, and Alexandr V. Ilyaskin

Supporting information Table of Contents

Figure S1; Figure S2; Figure S3; Figure S4; Figure S5; Figure S6; Figure S7; Figure S8; Figure S9; Figure S10; Figure S11; Figure S12; Figure S13.

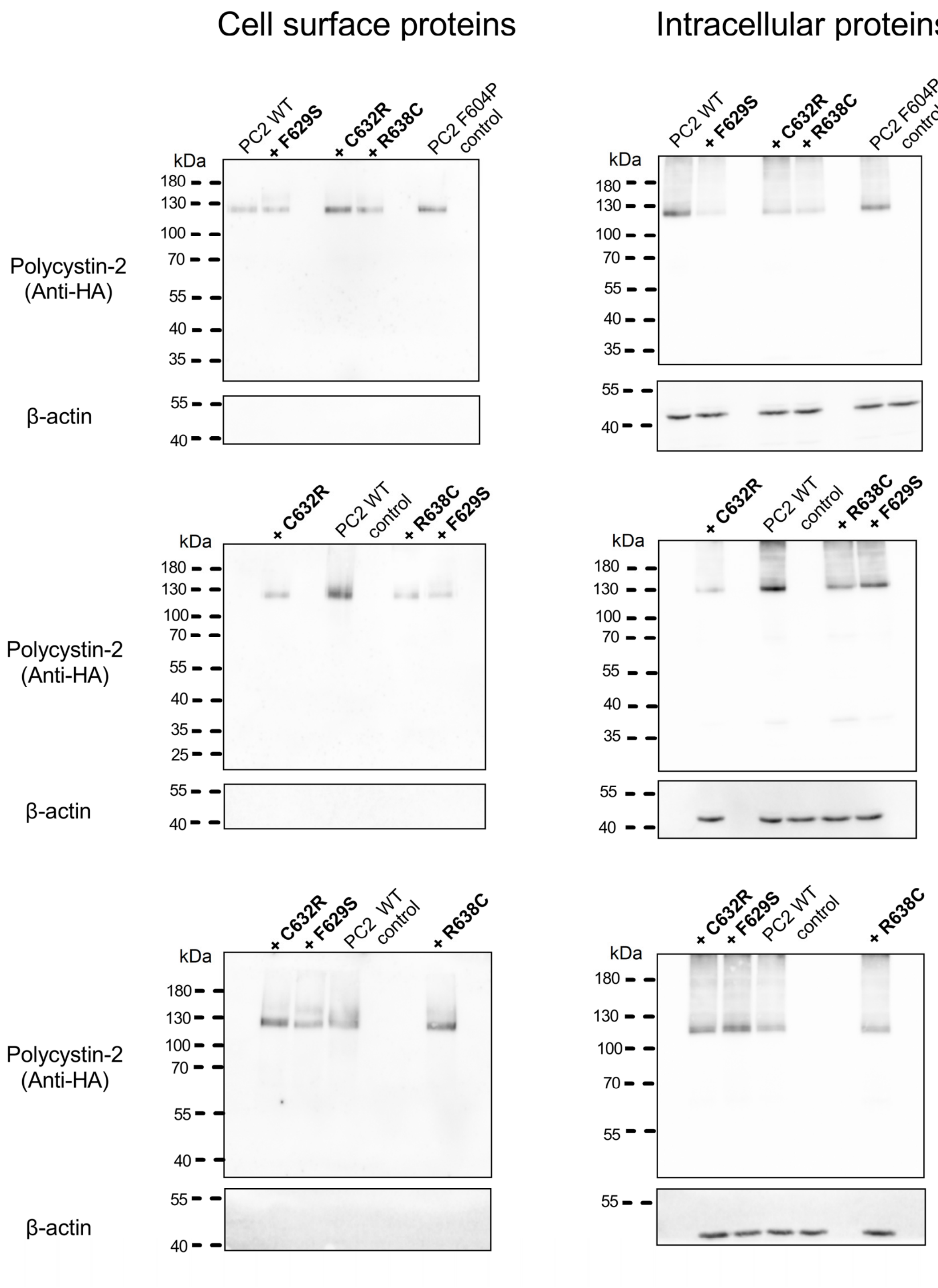

Figure S1: Western blot analysis of cell surface and intracellular expression of PC2 WT without and with F629S, C632R, R638C pore mutations.
Intracellular (*right panels*) or cell surface (*left panels*) expression of HA-tagged PC2 WT without or with a pore mutation as indicated (+F629S, +C632R or +R638C) from three different oocyte batches (N=3). No specific signal was detected with the anti-HA antibody in control oocytes. In addition, in the first batch of oocytes expression of HA-tagged PC2 F604P GOF construct was analyzed in parallel with other PC2 constructs. To validate separation of cell surface proteins from intracellular proteins, blots were stripped and re-probed using an antibody against β-actin.

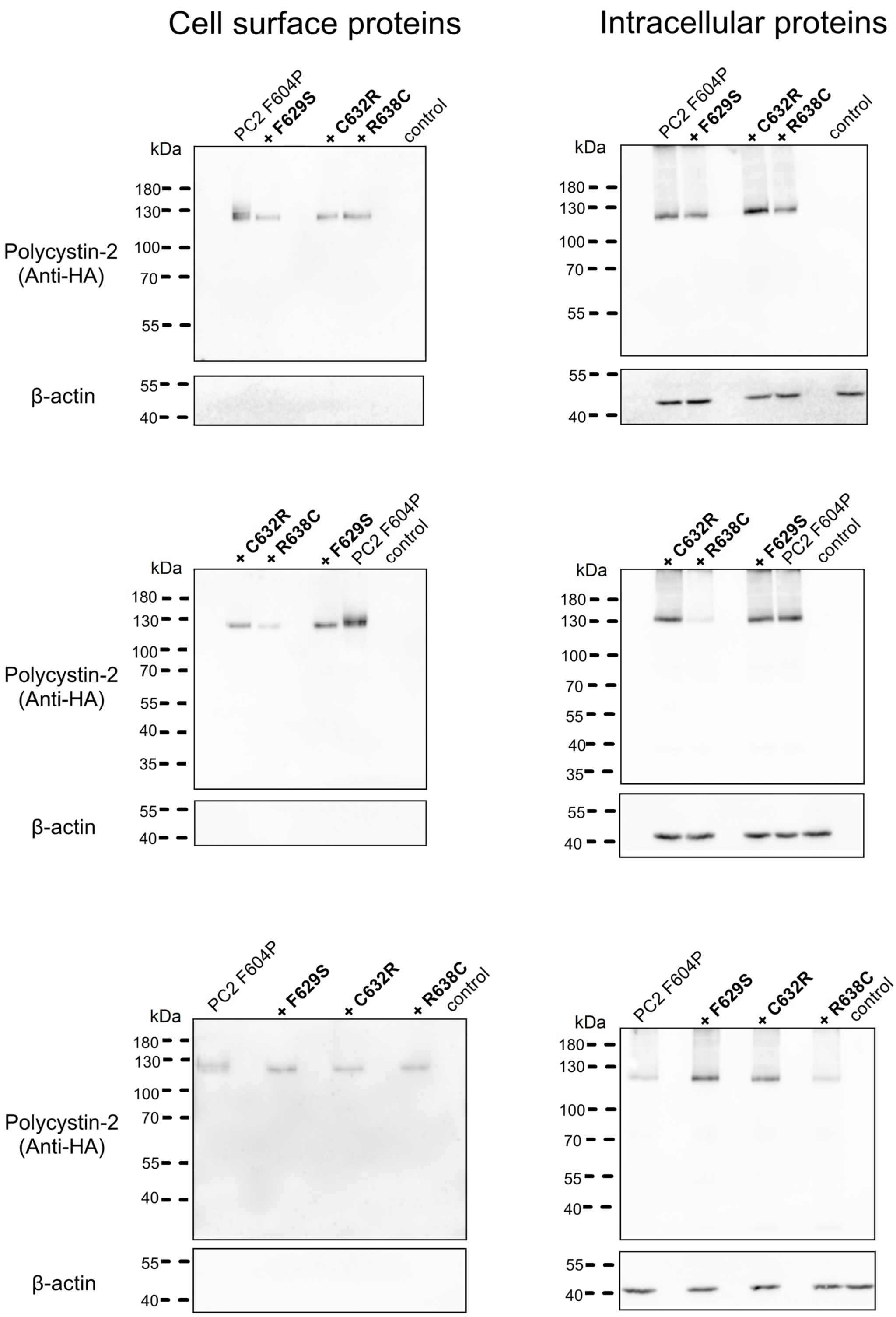

Figure S2: Western blot analysis of cell surface and intracellular expression of PC2 F604P without and with F629S, C632R, R638C pore mutations.

Intracellular (*right panels*) or cell surface (*left panels*) expression of HA-tagged PC2 F604P without or with a pore mutation as indicated (+F629S, +C632R or +R638C) from three different oocyte batches (N=3). No specific signal was detected with the anti-HA antibody in control oocytes. To validate separation of cell surface proteins from intracellular proteins, blots were stripped and reprobed using an antibody against β-actin.

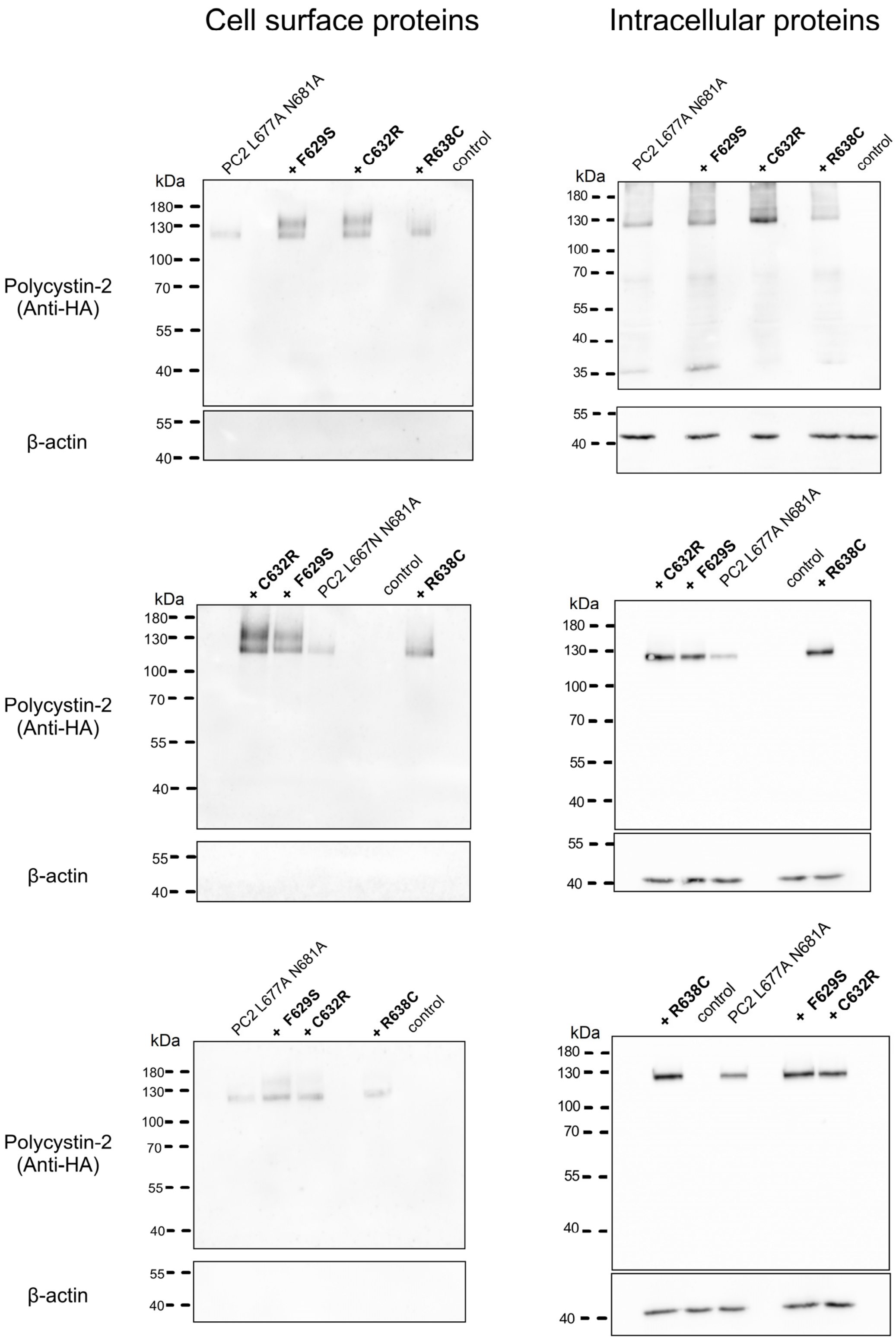

Figure S3: Western blot analysis of cell surface and intracellular expression of PC2 L677A N681A without and with F629S, C632R, R638C pore mutations.
Intracellular (*right panels*) or cell surface (*left panels*) expression of HA-tagged PC2 L677A N681A without or with a pore mutation as indicated (+F629S, +C632R or +R638C) from three different oocyte batches (N=3). No specific signal was detected with the anti-HA antibody in control oocytes. To validate separation of cell surface proteins from intracellular proteins, blots were stripped and reprobed using an antibody against β-actin.

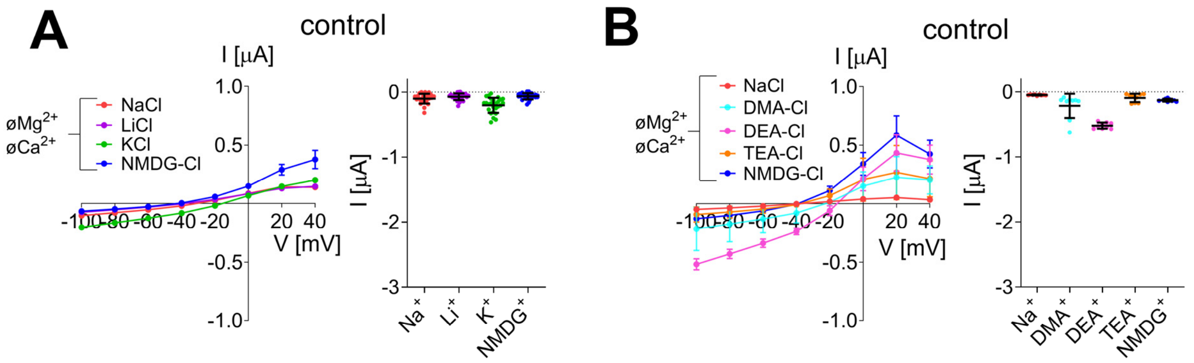

Figure S4: Effect of monovalent cation substitutions on baseline currents in control oocytes.

(**A**, **B**) *Left panels:* Average I/V-plot (mean ± SD) were obtained from control oocytes injected only with antisense DNA oligonucleotide against *Xenopus* connexin 38 (ASCx38) using a similar experimental protocol as described is Fig.6. *Right panels:* Summary data show the maximal inward currents reached during application of hyperpolarizing pulses of −100 mV in the presence of different monovalent cations in the bath as indicated. Measurements from individual oocytes and mean ± SD are shown (**A**: N=3, *n*=26; **B**: N=1, *n*=9; N indicates the number of different batches of *Xenopus laevis* oocytes, and *n* indicates the number of individual oocytes analysed per experimental group). In Fig. 6 these average whole-cell currents in different bath solutions were used to correct corresponding current values obtained in oocytes expressing PC2 constructs for endogenous oocyte currents. Despite this correction, the reversal potential measurements and the permeability ratios estimated from reversal potential shifts have to be interpreted with some caution.

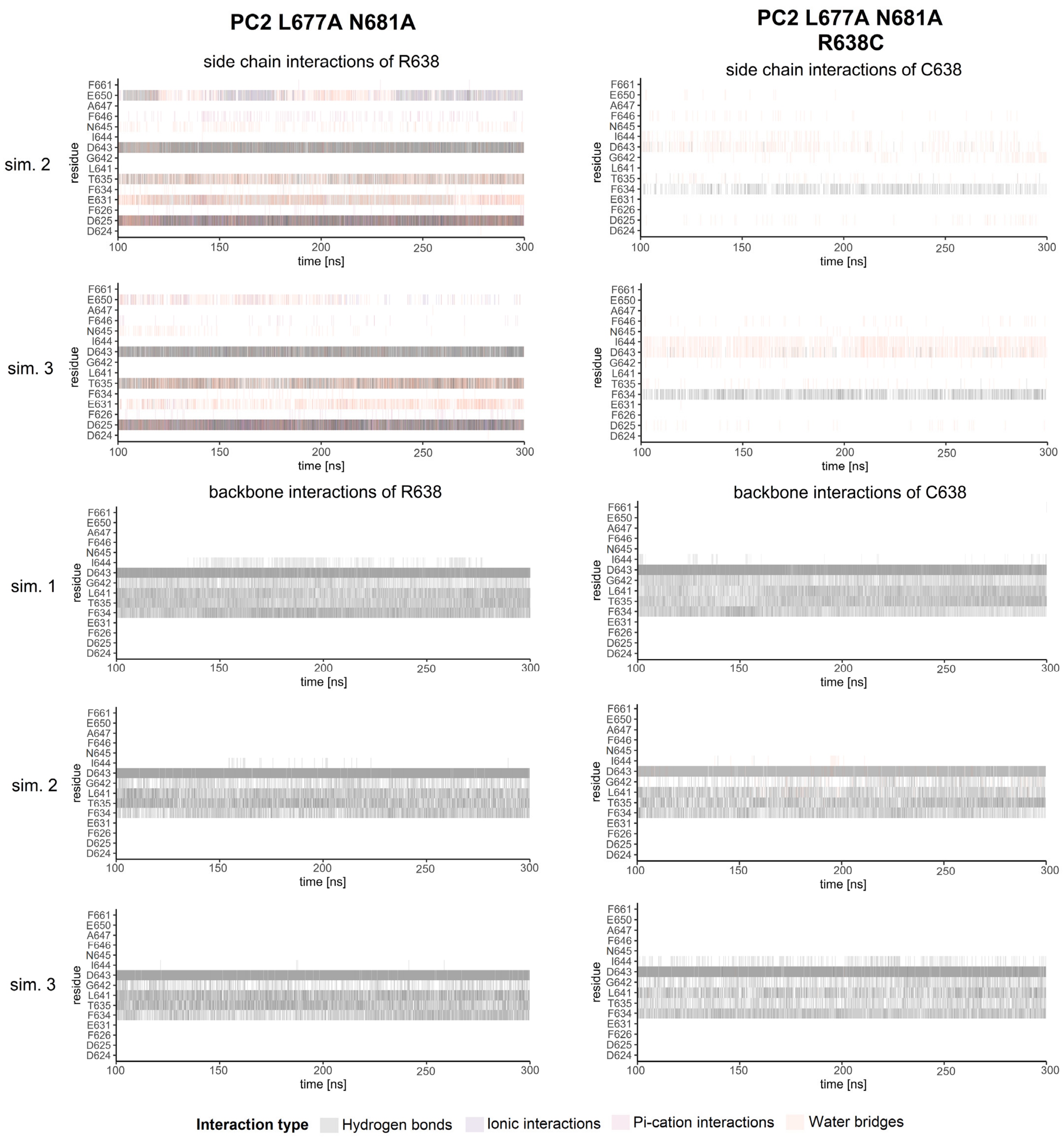

Figure S5: Analysis of side chain and backbone interactions of R638 or C638 in PC2 L677A N681A or PC2 L677A N681A R638C, respectively. Data were obtained over the last 200 ns of each replica MD simulation (sim. 1, sim. 2, sim.3). Side chain interactions observed in the first MD simulation are shown in Fig. 8C, D. Each vertical line represents the formation of at least one interaction of the specified type per trajectory frame. Interaction types are represented by different colors as indicated. Results from four PC2 subunits are pooled. A darker color intensity corresponds to multiple interactions of the same type observed at the same time point.

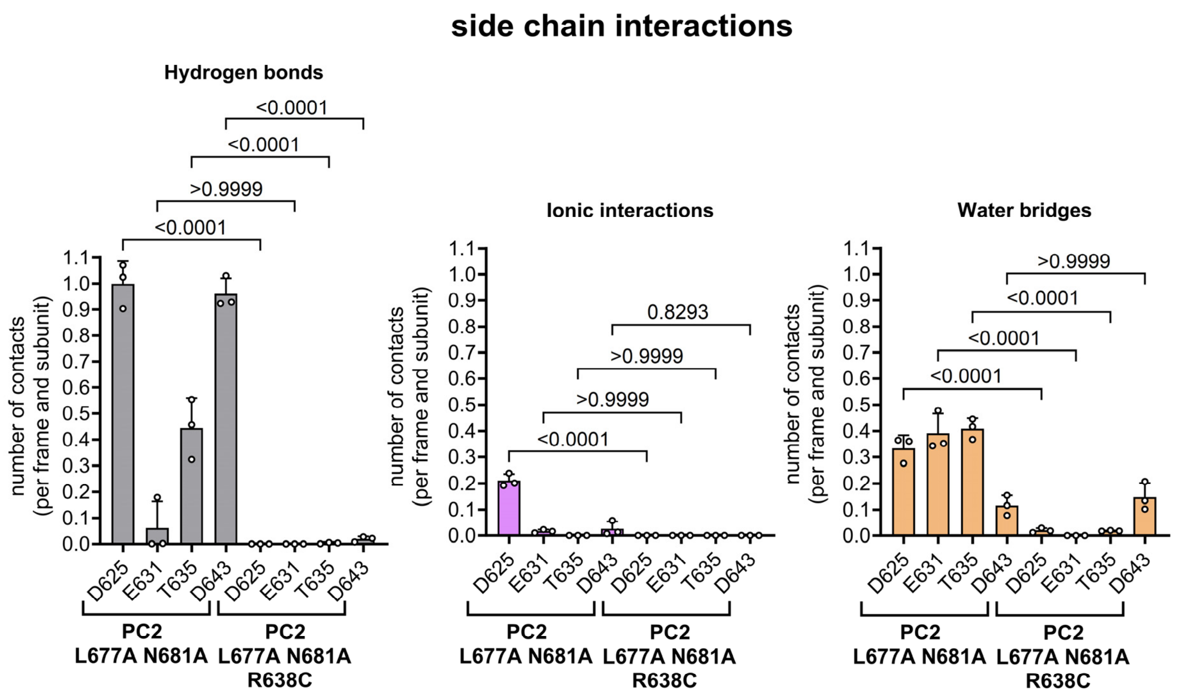

Figure S6: Quantification of interactions formed by the side chain of R638 or C638 with the four key pore loop residues. Summary data obtained from the same MD simulations as shown in Fig. 8C, D and Fig. S8. Each dot represents the average number of contacts formed by the side chain atoms of R638 (PC2 L677A N681A) or C638 (PC2 L677A N681A R638C) with an indicated pore loop residue over the last 200 ns of each MD simulation per trajectory frame and per PC2 subunit (n=3, the p-values were calculated by one-way ANOVA with Bonferroni post hoc test).

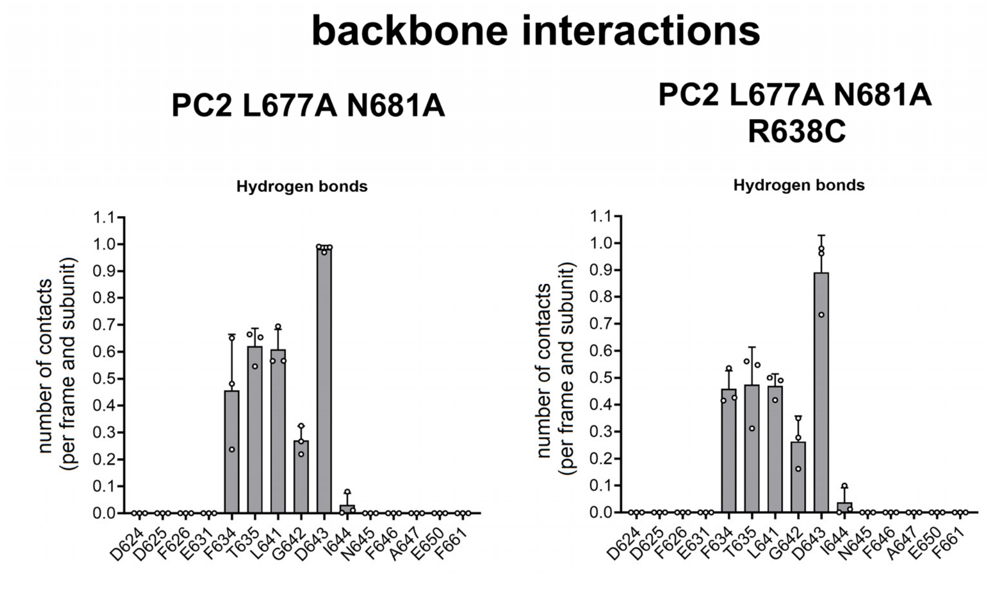

Figure S7: Quantification of interactions formed by the backbone of R638 or C638 with the pore loop residues. Summary data obtained from the same MD simulations as shown in Fig. S8. Each dot represents the average number of contacts formed by the backbone atoms of R638 (PC2 L677A N681A) or C638 (PC2 L677A N681A R638C) with an indicated pore loop residue over the last 200 ns of each MD simulation per trajectory frame and per PC2 subunit (n=3).

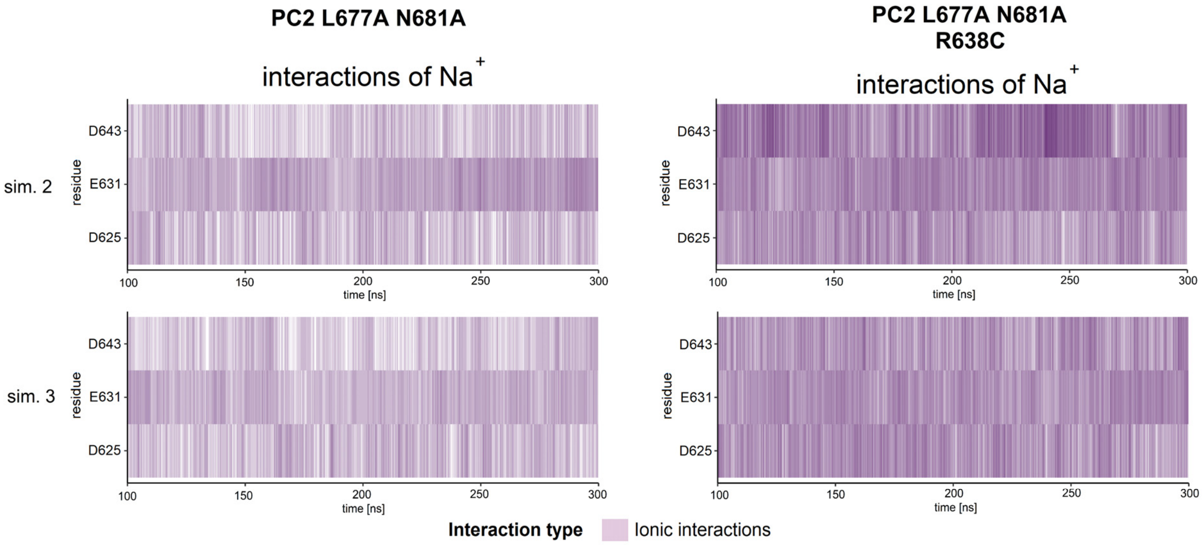

Figure S8: R638C mutation fosters ionic interactions between Na$^+$ and negatively charged pore loop residues. Data were obtained over the last 200 ns of each replica MD simulation (sim. 2, sim.3). Similar results were observed in the first MD simulation shown in Fig. 8E, F. Each vertical line represents the formation of at least one ionic interaction per trajectory frame. Results from four PC2 subunits are pooled. A darker color intensity corresponds to multiple interactions observed at the same time point.

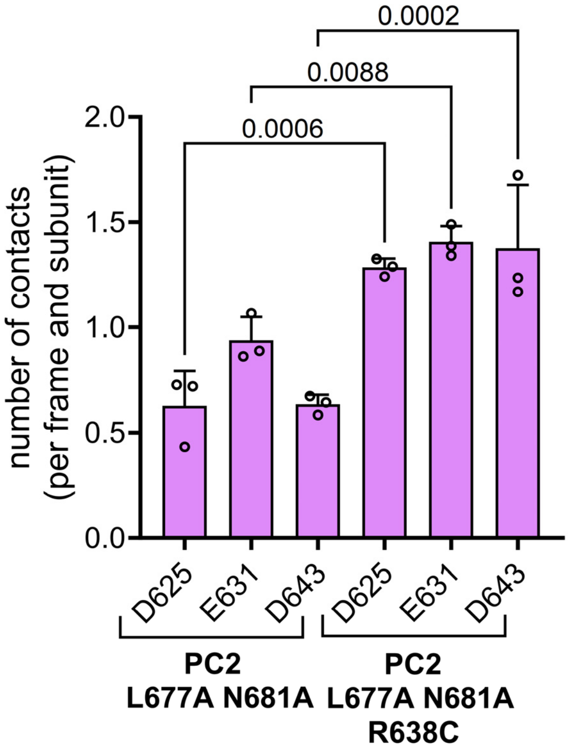

Figure S9: Quantification of interactions formed by Na$^+$ with the negatively charged pore loop residues. Summary data obtained from the same MD simulations as shown in Fig. 8 and Fig. S11. Each dot represents the average number of contacts formed by Na$^+$ with an indicated pore loop residue in PC2 L677A N681A or PC2 L677A N681A R638C over the last 200 ns of each MD simulation per trajectory frame and per PC2 subunit (n=3; the p-values were calculated by one-way ANOVA with Bonferroni post hoc test).

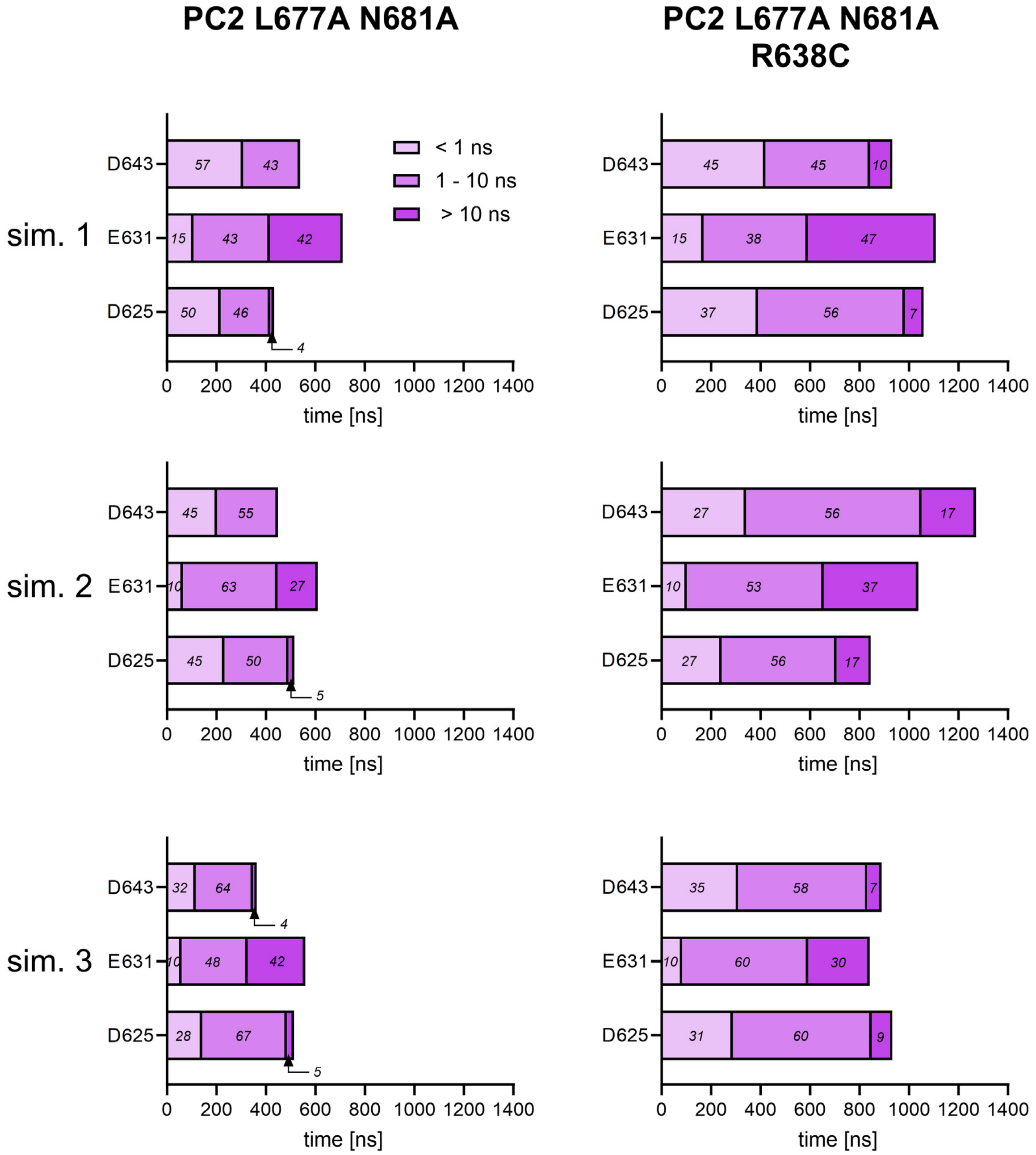

Figure S10: R638C substitution increases the duration of Na^+ interactions with negatively charged pore loop residues D625, E631 and D643.
Cumulative time of interactions between Na^+ ions and the indicated residues in all four PC2 subunits over the last 200 ns of the simulation time in three replica simulations (sim. 1, sim. 2, sim. 3) of PC2 L677A N681A without (*left panels*) or with pore mutation R638C (*right panels*). Duration of each interaction was estimated by multiplying the number of consecutive frames, in which this interaction was observed, by the sampling interval of the MD trajectory (sim. 1: 100 ps; sim. 2 and 3: 300 ps). More than one Na^+ can interact with a respective residue simultaneously, therefore the cumulative time of interactions may exceed the cumulative MD simulation time (4 subunits × 200 ns = 800 ns). Interactions are grouped by duration into three categories, 'short-term' (< 1 ns), 'medium-term' (1 – 10 ns) and 'long-term' (> 10 ns) interactions, and their respective proportions are shown by different colors and numbers inside bars (in % of total for each residue).

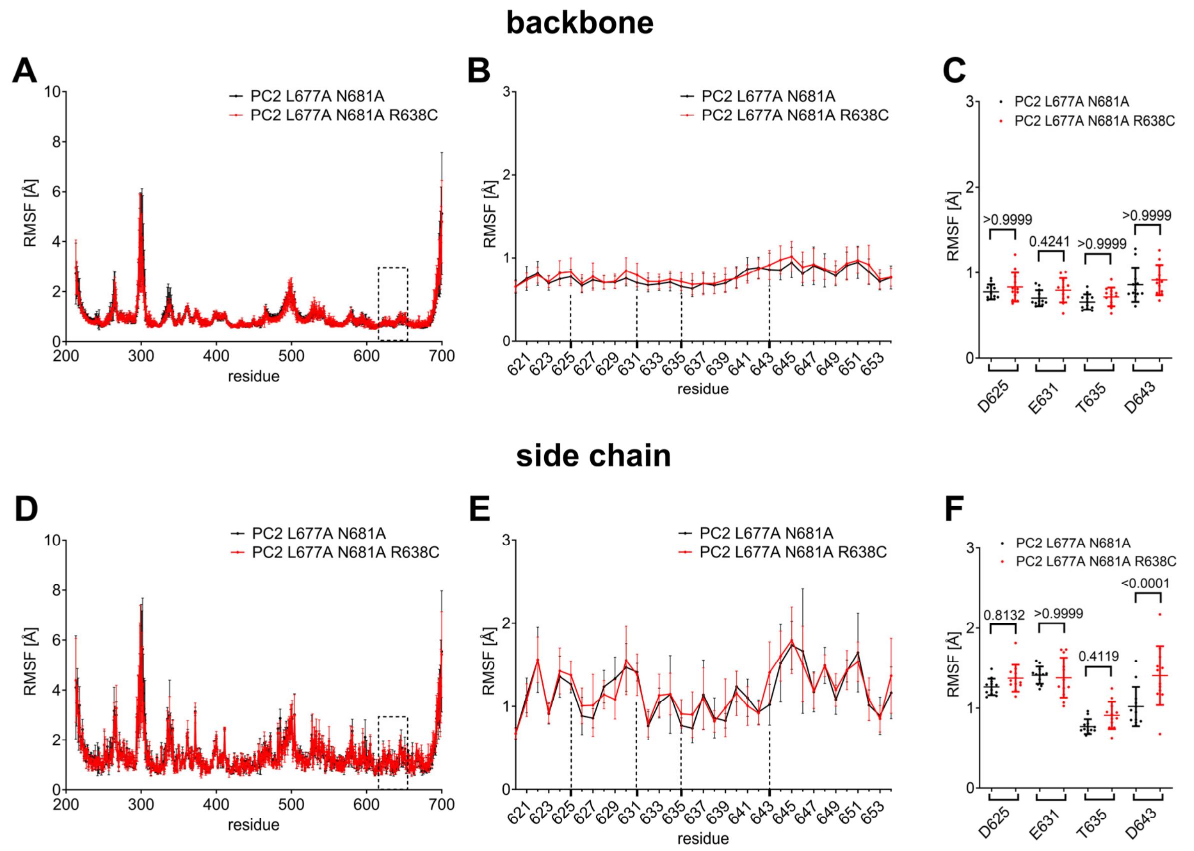

Figure S11: R638C substitution significantly increases root mean square fluctuation (RMSF) of D643 side chain. RMSF values were calculated for the side chain atoms (**A**-**C**) and backbone atoms (**D**-**F**) over the last 200 ns of each replica MD simulation. Values for the whole protein (**A**, **D**), for the pore loop (**B**, **E**; within the frame in **A**, **D**) and for the four key pore loop residues (**C**, **F**; indicated by vertical dotted lines in **B**, **E**) are shown (n=12: 4 subunits × 3 simulations; the p-values were calculated by one-way ANOVA with Bonferroni post hoc test).

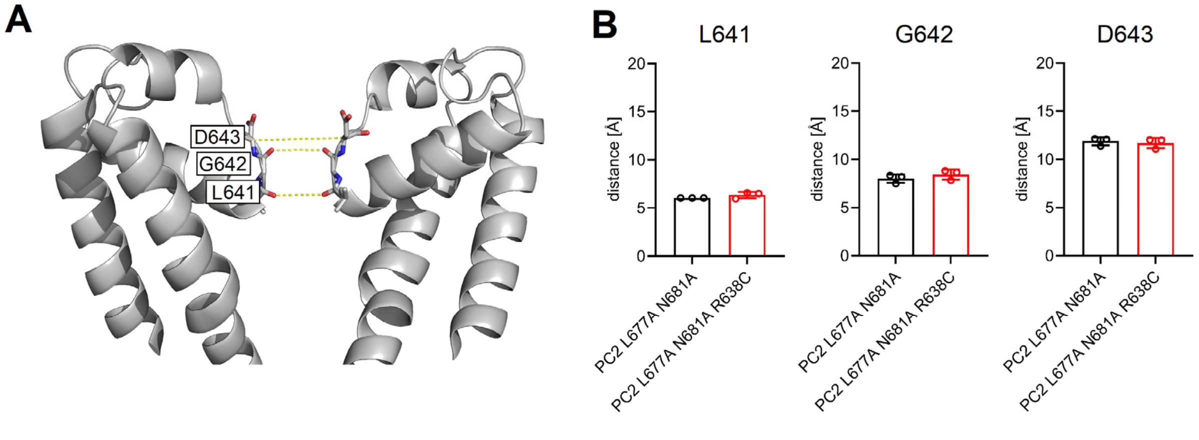

Figure S12: R638C mutation did not alter the dimensions of the channel's selectivity filter. **(A)** Pore loop regions of two diagonally opposed PC2 protomers in ribbon representation with three residues forming the selectivity filter (L641, G642 and D643) in sticks representation are shown. Distances between corresponding atoms of two subunits which are connected by yellow dotted lines were calculated and are shown in (**B**). Mean distances were calculated in each replica MD simulation (n=3).

PC2 L677A N681A
R638K

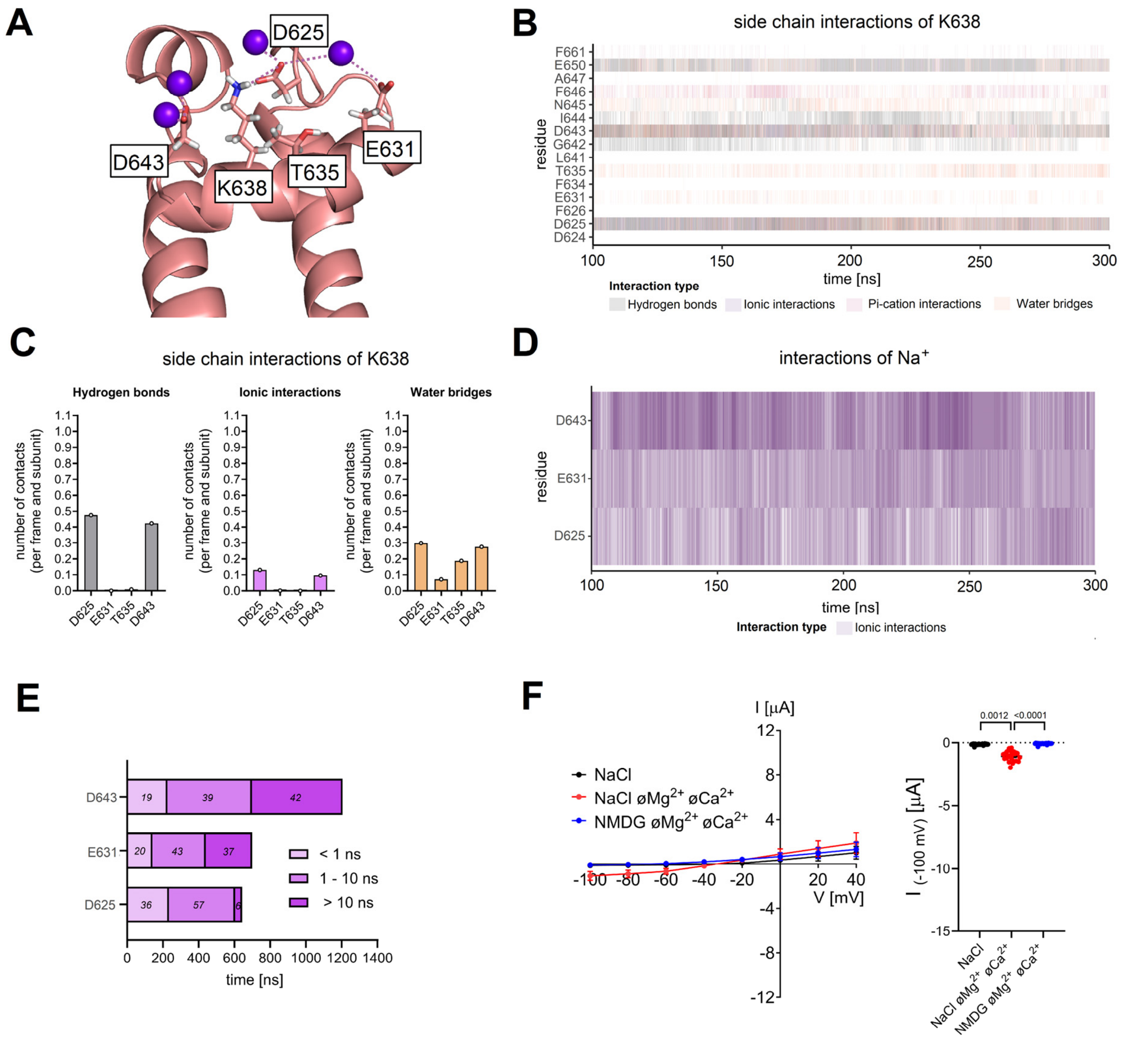

Figure S13: A conservative R638K substitution reduces Na$^+$ conductance of PC2 L677A N681A probably by disturbing the network of side chain interactions within the pore loop and increasing the number and duration of Na$^+$ interactions with D643

(**A**) Representative snapshot taken from an MD simulation of PC2 L677A N681A with R638K pore mutation shows ionic interactions of indicated residues within the pore loop with Na$^+$ and an ionic interaction between K638 and D625. Ribbon diagram of the channel's pore loop is shown with side chains of indicated residues in sticks representation, carbon atoms are in rose pink, hydrogen in white, oxygen in red, nitrogen in blue. Na$^+$ are represented as violet spheres. (**B**) Analysis of side chain interactions of K638 in PC2 L677A N681A R638K. Data were obtained over the last 200 ns of the MD simulation. Each vertical line represents the formation of at least one interaction of the specified type per trajectory frame. Interaction types are represented by different colors as indicated. Results from four PC2 subunits are pooled. A darker color intensity corresponds to multiple interactions of the same type observed at the same time point. (**C**) Quantification of interactions formed by the side chain of K638 with the four pore loop residues (D625, E631, T635, D643). Summary data was obtained from the same MD simulation as shown in (**B**). (**D**) Analysis of ionic interactions between Na$^+$ ions and the negatively charged pore loop residues (D625, E631, D643) was performed for the last 200 ns of the MD simulation. Individual interactions are represented with vertical violet lines. Results

from four PC2 subunits are pooled. Darker color intensity corresponds to multiple interactions observed at the same time point. (**E**) Cumulative time of interactions between Na^+ ions and the indicated residues in all four PC2 subunits over the last 200 ns of the simulation time. Quantification of the interaction time was performed as described in Figure S13. Interactions are grouped by duration into three categories, 'short-term' (< 1 ns), 'medium-term' (1 – 10 ns) and 'long-term' (> 10 ns) interactions, and their respective proportions are shown by different colors and numbers inside bars (in % of total for each residue). (**F**) *Left panel:* Average I/V-plot (mean ± SD) were obtained from oocytes expressing PC2 L677A N681A with pore mutation R638K using a similar experimental protocol as described is Fig. 2. *Right panel:* Summary data show the maximal inward currents reached during application of hyperpolarizing pulses of −100 mV in different bath solutions as indicated. Measurements from individual oocytes and mean ± SD are shown (N=2, *n*=23; N indicates the number of different batches of *Xenopus laevis* oocytes, and *n* indicates the number of individual oocytes analysed per experimental group; the p-values were calculated by Friedman test with Dunn's post hoc test).
